# YELLOW, SERRATED LEAF is essential for cotyledon vein patterning in Arabidopsis

**DOI:** 10.1093/plphys/kiae465

**Published:** 2024-09-03

**Authors:** Yetao Wang, Yutong Zheng, Yafei Shi, Deyuan Jiang, Qi Kuang, Xiangsheng Ke, Ming Li, Yukun Wang, Xiaohong Yue, Qin Lu, Xin Hou

**Affiliations:** State Key Laboratory of Hybrid Rice, Hubei Hongshan Laboratory, College of Life Sciences, Wuhan University, Wuhan 430072, China; State Key Laboratory of Hybrid Rice, Hubei Hongshan Laboratory, College of Life Sciences, Wuhan University, Wuhan 430072, China; State Key Laboratory of Hybrid Rice, Hubei Hongshan Laboratory, College of Life Sciences, Wuhan University, Wuhan 430072, China; State Key Laboratory of Hybrid Rice, Hubei Hongshan Laboratory, College of Life Sciences, Wuhan University, Wuhan 430072, China; State Key Laboratory of Hybrid Rice, Hubei Hongshan Laboratory, College of Life Sciences, Wuhan University, Wuhan 430072, China; State Key Laboratory of Hybrid Rice, Hubei Hongshan Laboratory, College of Life Sciences, Wuhan University, Wuhan 430072, China; State Key Laboratory of Hybrid Rice, Hubei Hongshan Laboratory, College of Life Sciences, Wuhan University, Wuhan 430072, China; State Key Laboratory of Hybrid Rice, Hubei Hongshan Laboratory, College of Life Sciences, Wuhan University, Wuhan 430072, China; State Key Laboratory of Hybrid Rice, Hubei Hongshan Laboratory, College of Life Sciences, Wuhan University, Wuhan 430072, China; State Key Laboratory of Hybrid Rice, Hubei Hongshan Laboratory, College of Life Sciences, Wuhan University, Wuhan 430072, China; State Key Laboratory of Hybrid Rice, Hubei Hongshan Laboratory, College of Life Sciences, Wuhan University, Wuhan 430072, China

## Abstract

Venation develops complex patterns within the leaves of angiosperms, and the mechanism of leaf vein patterning remains poorly understood. Here, we report a spontaneous mutant that exhibits yellow serrated leaves and defective cotyledon vein patterning. We mapped and cloned the relevant gene *YELLOW*, *SERRATED LEAF* (*YSL*), a previously unreported gene in plants. YSL interacts with VH1-interacting kinase (VIK), a protein that functions in cotyledon venation development. VIK is a vascular-specific adaptor protein kinase that interacts with another vascular developmental protein, VASCULAR HIGHWAY1 (VH1)/BRASSINOSTEROID INSENSITIVE 1-LIKE 2 (BRL2), which is a receptor-like kinase of the BRASSINOSTEROID INSENSITIVE 1 (BRI1) family. Mutation of *YSL* affects the auxin response and the expression of auxin-related genes in Arabidopsis (*Arabidopsis thaliana*). Our results reveal that YSL affects cotyledon vein patterning by interacting with VIK in *Arabidopsis*.

## Teaser

YELLOW, SERRATED LEAF interacts with a vascular-specific adaptor protein and regulates cotyledon vein patterning via modulating the auxin response in Arabidopsis.

## Introduction

The plant vascular network carries out essential functions in plant growth and leaf development. It provides mechanical support for plants, and the proper structure of the vascular system ensures the translocation of water, essential mineral nutrients, sugars, and amino acids to plant organs (Lucas et al. 2013; Furuta et al. 2014; Biedron and Banasiak 2018). The model plant Arabidopsis (*Arabidopsis thaliana*) has a simple structure, and its cotyledon is an ideal object for studying vein patterning. Before the first true leaves emerge, cotyledons provide energy for plant growth through photosynthesis (Liu et al. 2022). The *Arabidopsis* cotyledon vein pattern consists of the midvein (primary vein) and secondary veins. The midvein forms from the petioles to the apex of the cotyledon. The midvein-linked secondary veins normally form 4 areoles (Sieburth 1999).

Several Arabidopsis leaf or cotyledon vein patterning defects, including reduced vein number, discontinuous veins, and additional loops, have been reported. Mutations in *AUXIN-RESISTANT6* (*AXR6*), *MONOPTEROS* (*MP*) and *BODENLOS* (*BDL*) resulted in reduced leaf vein (Hobbie et al. 2000; Garrett et al. 2012). Loss of function of *COTYLEDON VASCULAR PATTERN 1* (*CVP1*), *COTYLEDON VASCULAR PATTERN 2* (*CVP2*), *VASCULAR NETWORK DEFECTIVE 1* to *7 VAN1*-*7* and *SCARFACE SFC* results in a discontinuous leaf vein pattern (Carland et al. 1999; Deyholos et al. 2000; Koizumi et al. 2000; Carland and Nelson 2004). Mutation of *At5PTase13* results in abnormal cotyledon vein patterning, which includes altered loops, branches, intersections, and fusions of the distal and proximal secondary veins (Lin et al. 2005). The VASCULAR HIGHWAY1 (VH1)/BRI1-LIKE 2 (BRL2) is a provascular cell type-specific receptor kinase, and functional loss of the *VH1* gene results in premature leaf senescence and defective vascular transport (Clay and Nelson 2002). Ectopic expression of *VH1* in *Arabidopsis* plants affects venation patterning and cell proliferation (Clay and Nelson 2002). VH1-interacting kinase (VIK) can interact with VH1 to influence leaf venation. Mutation of *VH1* or *VIK* results in a defective pattern of cotyledon veins where secondary veins are unconnected apically to the primary vein (midvein) or interrupted by a gap (Ceserani et al. 2009). VH1/BRL2 and VIK influence auxin and brassinosteroid signaling and play a part in establishing vein patterns in leaves.

During the growth and development of leaves, small outgrowths, serrations, or leaflets may form in marginal regions (Efroni et al. 2010). Different division rates and growth rates of marginal cells result in serration in leaf margins. Compared with the entire leaf, the serrated or lobed leaves provide a larger leaf area, which more easily absorbs light energy (Semchenko and Zobel 2007). In addition, the shape of the leaf margin can regulate the temperature of the blade surface and water runoff, and the leaf margins are superior in terms of heat dissipation and cold tolerance (Vogel 2009).

Auxin plays an essential role in cotyledon vein formation and leaf marginal morphology. There are 2 theories about the role of auxin in leaf vascular development: reaction–diffusion prepattern and auxin signal flow canalization (Nelson and Dengler 1997; Merks et al. 2007). The first theory holds that interactions between several morphogens locally determine vein patterning. The second theory holds that auxin flow forms narrow strands, vein pattern formation occurs along the apical–basal pathways of auxin flow. Vascular tissues differentiate in regions where the auxin concentration is elevated. The occurrence of leaf serration also associates with the presence of auxin. Auxin maxima were present at the tip of the leaf serration, and the auxin content was lower in the sinus regions. The accumulation of the auxin efflux carrier PIN-FORMED 1 (PIN1) led to auxin maxima, which was reinforced by auxin feedback from PIN1 (Bilsborough et al. 2011; Byrne 2012). CUP-SHAPED COTYLEDON2 (CUC2) is a key component in leaf serration. CUC2 can direct PIN1 accumulation. CUC2 activity is needed for the production of the auxin maxima (Bilsborough et al. 2011; Byrne 2012). Several proteins related to leaf vein development have been reported to be related to auxin. For example, MP participates in the regulation of auxin-induced gene expression (Hardtke and Berleth 1998; Garrett et al. 2012; Krogan et al. 2012). *scarface* (*sfc*) plants show altered auxin transport and response (Sieburth et al. 2006). Mutation of *At5PTase13* alters the expression of genes related to auxin biosynthesis and transport (Lin et al. 2005). VH1/BRL2 and VIK influence auxin signaling (Ceserani et al. 2009).

In this study, we identified a spontaneous mutant in *Arabidopsis* with yellow, serrated leaves and a defective cotyledon vein pattern and named it *yellow*, *serrated* leaves (*ysl*). Then, we cloned the relevant gene *YSL* by genetic mapping and bulked segregant analysis (BSA). *YSL* encodes a homolog of the human CCDC25 protein but has not been characterized in plants. *YSL* is highly expressed in actively dividing regions of leaves (cotyledon vein, leaf apex, and apical meristem). YSL interacts with the vascular-specific adaptor protein VIK, which is related to the cotyledon vein pattern. Deficiency of YSL reduced the response of plants to auxin and the expression of several auxin-related genes in *Arabidopsis*. These results demonstrated that *YSL* plays a critical role in cotyledon vein patterning in *Arabidopsis*.

## Results

### Identification of a yellow and serrated *Arabidopsis* mutant

**Figure 1.**
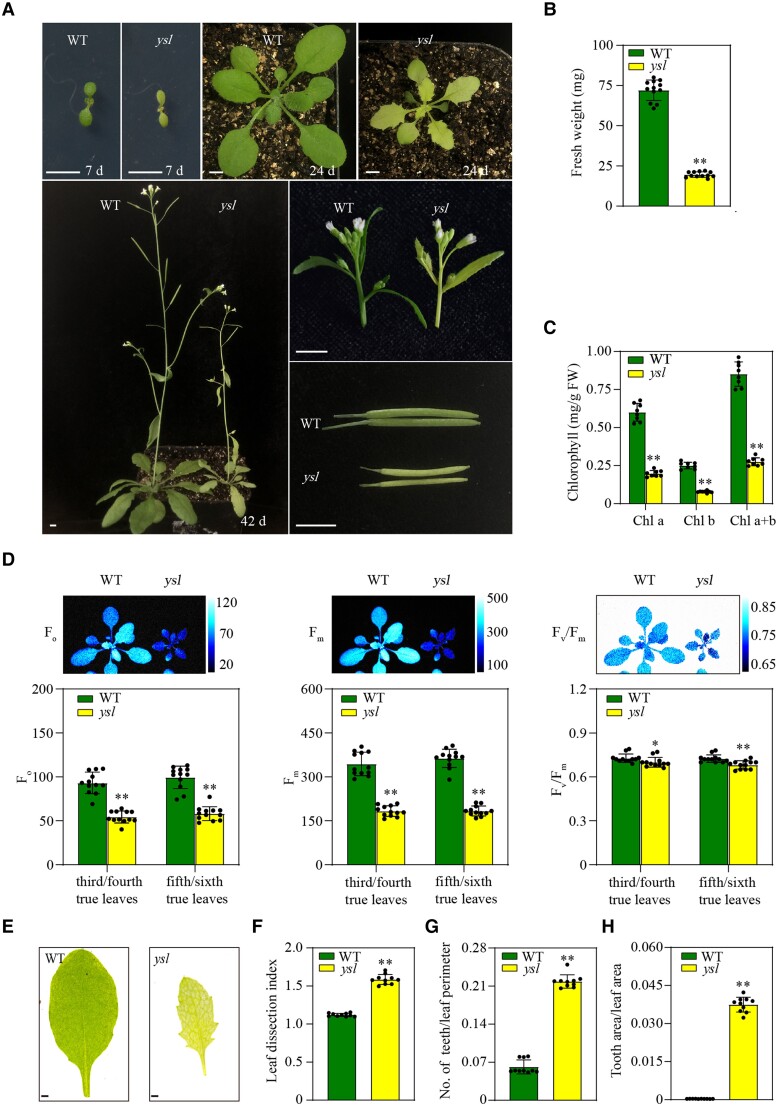
Phenotypic characterization of *ysl* mutant plants. **A)** Phenotypes of wild type (WT) and *ysl* plants at different growth stages. Scale bars: 0.5 cm. **B)** Fresh weight (FW) of 24-d-old WT and *ysl* plants. The bars indicate the mean ± SD (*n* = 10). **Student's *t*-test with *P* < 0.01. Three biological replicates were performed. **C)** Chlorophyll content of 24-d-old WT and *ysl* plants. The bars indicate the mean ± SD (*n* = 8). **Student's *t*-test with *P* < 0.01. Three biological replicates were performed. **D)** Chlorophyll fluorescence of 24-d-old WT and *ysl* plants. The chlorophyll fluorescence parameters of the third/fourth and fifth/sixth true leaves of the WT and *ysl* plants were calculated. *F*_o_, minimal fluorescence; *F*_m_, maximal fluorescence; *F*_v_/*F*_m_, maximum efficiency of PSII photochemistry. The bars indicate the mean ± SD (*n* = 12). *Student's *t*-test with *P* < 0.05; **Student's *t*-test with *P* < 0.01. Three biological replicates were performed. **E)** The fifth/sixth true leaves of 21-d-old WT and *ysl* plants. Scale bars: 1 mm. **F** to **H)** Quantitative comparisons of leaf shapes in WT and *ysl* plants based on the leaf dissection index (perimeter^2^/4π × leaf area) **F)**, the number of teeth/leaf perimeters **G)**, and the tooth area/leaf area **H)**. The bars indicate the mean ± SD (*n* = 10). **Student's *t*-test with *P* < 0.01.

We found a spontaneous mutant whose leaves were yellow and serrated at different growth stages, this mutant included cotyledons (7-d-old) and rosette leaves (24-d-old and 42-d-old) in Fig. 1A. Therefore, we named it *yellow*, *serrated leaf* (*ysl*). The cauline leaves, stems, young siliques, and flower clusters were also yellow (Fig. 1A). The fresh weight of the *ysl* mutants was lower than that of the WT plants among the 24-d-old plants (Fig. 1B). Since the level of chlorophyll is one of the most important indicators of leaf color, we assessed the chlorophyll content in *ysl* and WT plants. Consistent with the visible yellow leaf phenotype, the concentration of chlorophyll was substantially lower in *ysl* compared to WT plants (Fig. 1C). A decrease in chlorophyll content often leads to decreased photosynthetic capacity. Hence, we measured the chlorophyll fluorescence of 24-d-old WT and *ysl* plants. There was a reduction in photosynthetic parameters, including *F*_o_ (minimal fluorescence), *F*_m_ (maximal fluorescence) and *F*_v_/*F*_m_ (the maximum quantum yield of photosystem II), in the mutants (Fig. 1D), indicating that their photosynthetic capacity was impaired. Since leaf margin development was perturbed in *ysl* mutants, we analyzed the leaf shape of the fifth/sixth true leaves (Fig. 1E) based on 3 different parameters: the leaf dissection index (perimeter^2^/4π× leaf area), the number of teeth/leaf perimeters, and the tooth area/leaf area (Royer et al. 2008; Bilsborough et al. 2011). These parameters increased in *ysl* mutants (Fig. 1, F to H), which indicated that the mutation promoted outgrowth at the leaf margin.

### *YSL* encodes an unknown protein

To clone the relevant gene, a cross was performed to first determine the segregation of the *YSL* locus. We crossed *ysl* with Col-0. The F_1_ plants exhibited the same phenotype as the WT plants. The segregation based on 486 F_2_ plants fit at a ratio of 3:1 (χ^2^ = 0.5759<χ^2^_0.05,1_ = 3.84) (Table 1), indicating that the mutant phenotype was controlled by a single recessive nuclear gene.

**Table 1.** Chi-square test for segregating yellow and serrated leaves

Phenotypic classes	Observed value	Expected value	χ^2^ value (3:1)
Wild type	1327	1313.25	0.5759
Mutant	424	437.75	
Total	1751	1751	

**Figure 2.**
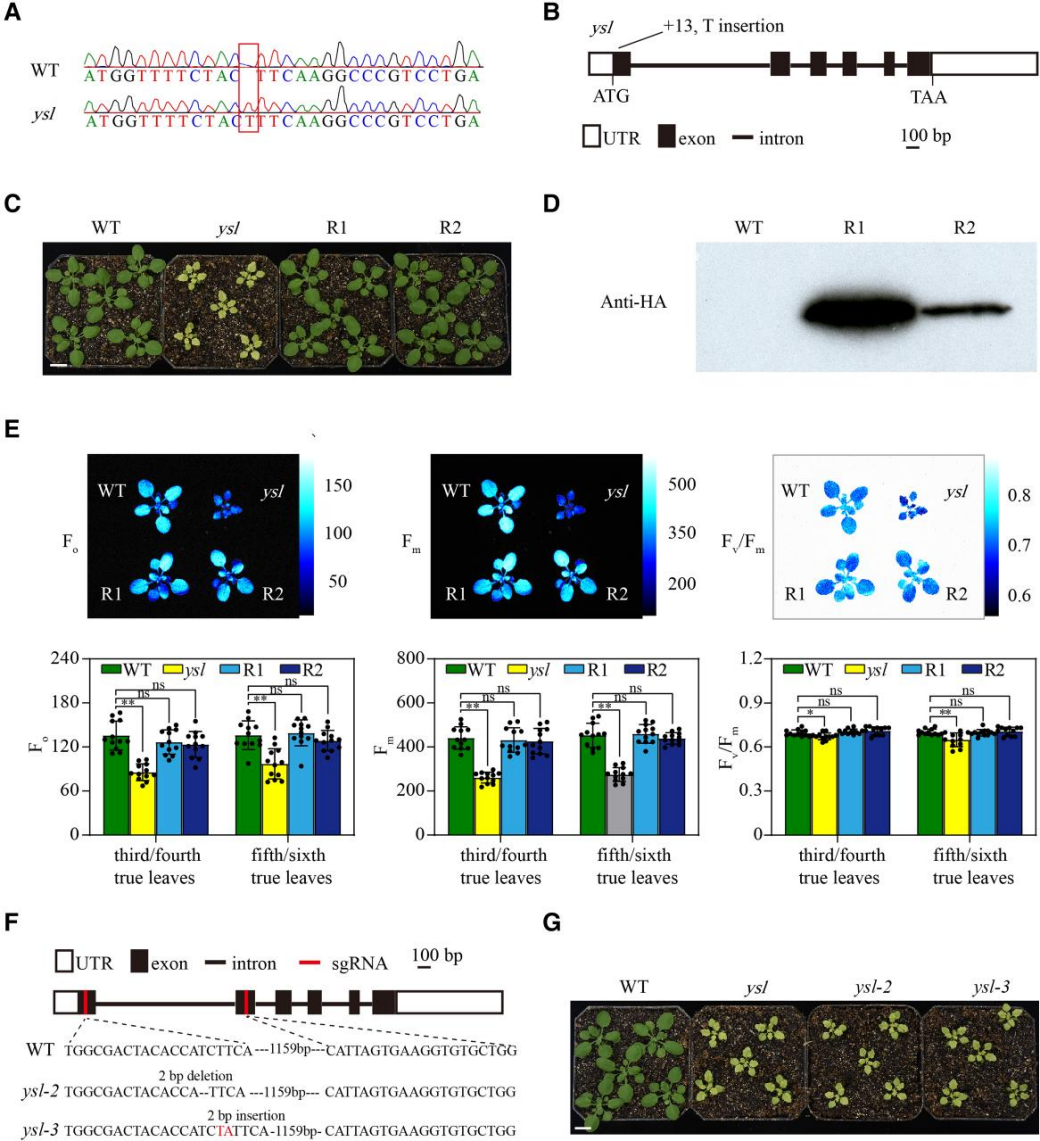
Cloning of the *YSL* gene. **A** to **J)** Sequence of the mutated region of the *YSL* gene (*AT5G11500*) in the wild type (WT) and *ysl*. Red boxes represent the mutation site. **B)** Gene structure of the *YSL* gene with the start codon (ATG) and stop codon (TAA) indicated. **C)** Rescue of *ysl*. The WT, *ysl*, and 2 rescued line (R1, R2) plants were grown in soil for 21 d. Scale bar: 1 cm. **D)** YSL protein expression in the rescued lines was examined by immunoblotting using an anti-HA antibody. **E)** Chlorophyll fluorescence of 24-d-old WT, *ysl*, and rescued plants. The chlorophyll fluorescence parameters of the third/fourth and fifth/sixth true leaves of WT and *ysl* plants were calculated. *F*_o_, minimal fluorescence; *F*_m_, maximal fluorescence; *F*_v_/*F*_m_, maximum efficiency of PSII photochemistry. The bars indicate the mean ± SD (*n* = 12). *Student's *t*-test with *P* < 0.05; **Student's *t*-test with *P* < 0.01. Three biological replicates were performed. **F)** Construction and identification of additional *ysl* alleles. Two knockout lines *ysl-2* (TC deletion at +47 bp) and *ysl-3* (TA insertion at +49 bp), were generated via the CRISPR-Cas9 method. sgRNA, small guide RNA. **G)** Consistent with *ysl*, *ysl-2*, and *ysl-3* exhibited yellow and serrated leaf phenotypes. Scale bar: 1 cm.

We used map-based cloning to isolate the candidate gene and locate the *YSL* locus between 2 simple sequence length polymorphism (SSLP) markers, M21 and M22, with exchange rates (Supplementary Table S1) of 0.04 and 0.17 on chromosome 5, respectively (Supplementary Fig. S1A). According to the crossover ratio, the *YSL* gene was mapped close to the first marker, M21 (Supplementary Fig. S1A). Subsequently, BSA was used for fine mapping. Whole-genome sequencing and BSA analysis revealed that the mutation was located at 1.5 to 5.5 Mb on chromosome 5 (Supplementary Fig. S1B). This was consistent with rough mapping. In this region, there were 22 candidate genes, and polymorphisms were located within the gene or upstream of the gene (Supplementary Table S2). Among these candidate genes, there was a T-insertion at the +13 bp nucleotide position of *At5g11500*, causing a frameshift in the encoded protein, which was subsequently confirmed by Sanger sequencing of the mutant site (Fig. 2, A and B).

To confirm that the phenotype of the *ysl* mutant was caused by this frameshift mutation in the *At5g11500* gene, we performed a rescue experiment. The complete 645 bp CDS (excluding the stop codon) of *At5g11500* was introduced into the expression vector (35S promoter, YFP, and HA tag) and ultimately transformed into mutants. The expression of the fusion protein completely rescued the mutant phenotypes (Fig. 2C). Immunoblotting results showed that a protein located at approximately 50 to 70 kDa was detected by using an HA antibody in the transgenic lines, which was consistent with the predicted size of the YSL-YFP-HA fusion protein (Fig. 2D). The expression of the fusion protein in *ysl* completely rescued the mutant phenotypes. We measured the chlorophyll fluorescence of 24-d-old WT, *ysl*, and rescued plants. In the rescued plants, the photosynthetic parameters, including *F*_o_, *F*_m_, and *F*_v_/*F*_m_, were restored to the same levels as those in the WT plants (Fig. 2E). These findings indicated that we identified the correct gene. Moreover, we knocked out the *AT5G11500* gene in WT plants using a CRISPR-Cas9 genome editing system (Xie et al. 2015) and obtained 2 additional *ysl* alleles. There was a TC deletion at +47 bp in *ysl-2* and a TA insertion at +49 bp in *ysl-3* (Fig. 2F). The phenotypes of these knockout lines (*ysl-2* and *ysl-3*) resembled those of *ysl* mutants (Fig. 2G). These results confirmed that the yellow and serrated leaf phenotypes were caused by a loss-of-function mutation in the *YSL* gene.

The homologous proteins of YSL are ubiquitous in eukaryotes. Aligning the homologous sequence in different species, we found that the *YSL* gene is conserved in rice, maize, barley, nematodes, zebrafish, humans, and rats (Supplementary Fig. S2A). The homolog of *YSL* has been reported only in humans. In humans, the homologous protein CCDC25 is a transmembrane protein that acts as a receptor for NET-DNA (the DNA component of neutrophil extracellular traps, which consists of chromatin DNA filaments coated with granule proteins, are released by neutrophils to trap microorganisms) on the cell membrane of cancer cells (Yang et al. 2020). According to the evolutionary analysis of homologous proteins in 30 representative species, homologous proteins exist in fungi, protists, plants, and animals, and their amino acid sequences are conserved (Supplementary Fig. S2B). These suggest that *YSL* originated from an ancient gene whose function was relatively important, and it was preserved during evolution.

### YSL is expressed in regions with active cell division and *is* located in the nucleus and cell membrane

**Figure 3.**
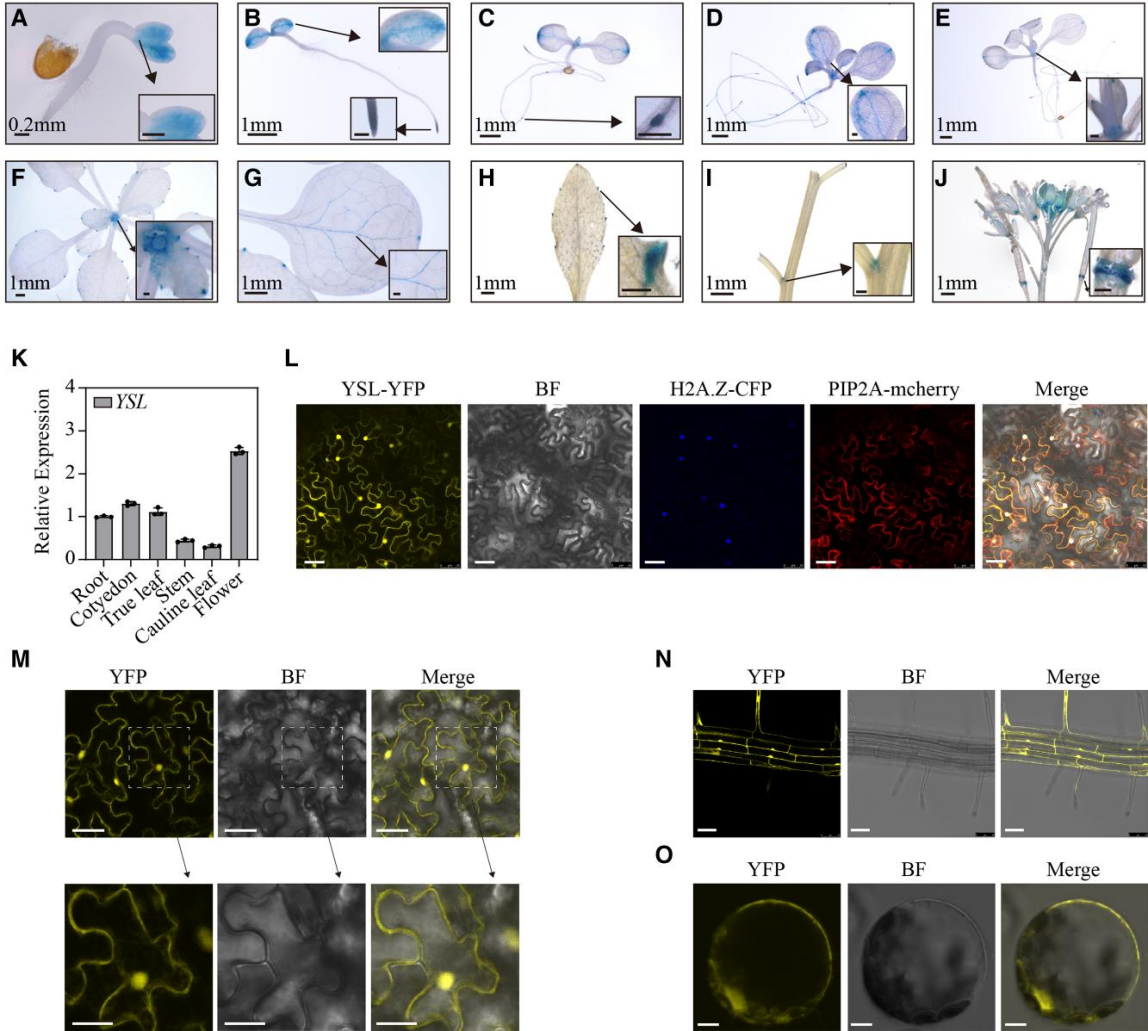
Expression patterns and localization of YSL. **A** to **J)** Expression patterns of *YSLpro::GUS* in transformed *Arabidopsis* plants at different growth stages. GUS staining of *YSLpro::GUS* transgenic plants showing *YSL* expressed in 2-d-old plants **A)**, 5-d-old plants **B)**, 6-d-old plants **C)**, 10-d-old plants **D)**, 12-d-old plants **E)**, 23-d-old plants **F)**, 23-d-old plants **G)**, cauline leaves of 37-d-old plants **H)**, stems of 37-d-old plants **I)**, and buds, flowers, and siliques of 50-d-old plants **J)**. Scale bars in enlarged image: 0.2 mm. **K)** Relative mRNA levels of *YSL* in different tissues (roots, cotyledons, true leaves, stems, and flowers) of 35-d-old WT plants grown in soil. The expression levels were determined using RT-qPCR. *ACTIN2* was used as an internal standard. **L)** Confocal microscopy images of the YSL-YFP fusion protein transiently coexpressed with marker proteins in *N. benthamiana* leaves. The H2A. Z-CFP is a nuclear marker, and PIP2A-mCherry is a cell membrane marker. Scale bars: 50 *μ*m. **M** to **O)** Confocal microscopy images of the YSL-YFP fusion protein expressed in the leaves **M)**, roots **N)**, and protoplasts **O)** of the rescued plants. Scale bars: 25 *μ*m (leaf), 12.5 *μ*m (enlarged leaf); 50 *μ*m (root); 7.5 *μ*m (protoplast).

To estimate the expression pattern of the *YSL* gene in *Arabidopsis*, we generated transgenic plants expressing the GUS reporter under the control of the hypothetical *YSL* promoter (a 2.0 kb fragment upstream of the start codon). GUS staining analysis revealed that the *YSL* promoter was active mainly in parts of the plant where cells were actively dividing, including the vein, leaf apex, root apex, and shoot meristem (Figure 3, A to J). With the growth of the seedlings, we observed high expression in the leaf apex, apical meristem, and lateral root primordia (Fig. 3, C to F). We also observed expression in the rosette leaf veins (Fig. 3G). When the plants bolted, we detected high GUS expression in the leaf apex and at the serration tips of the cauline leaf (Fig. 3H), stem lateral meristem (Fig. 3I), and flower (Fig. 3J). In siliques, high GUS expression was observed at the region of floral organ abscission (Fig. 3J). Consistent with the gus staining results, reverse transcription quantitative PCR (RT-qPCR) analysis revealed that *YSL* was expressed in the roots, cotyledons, true leaves, stems, cauline leaves, and flowers (Fig. 3K). The high activity of the *YSL* promoter in the cotyledon leaf vein, leaf apex and serration tip suggested that YSL may function in these regions. The functions of a gene can be deduced from its subcellular localization. In transient transformation experiments in *Nicotiana benthamiana*, we conducted protein colocalization analysis using nuclear and cell membrane markers along with the YSL-YFP fusion protein. The results showed that YSL was located in the nucleus and cell membrane (Fig. 3L). Additionally, we examined the leaves (Fig. 3M) and roots of the rescued plants (Fig. 3N) via confocal microscopy. The results were consistent with the location in *N. benthamiana*. Due to the presence of cell walls in both leaves and roots, we observed the protoplasts of the leaves of the rescued plants. The YFP signal was in the nucleus and cell membrane (Fig. 3O). These results indicated that YSL primarily localized to the nucleus and cell membrane.

### *YSL* mutation results in abnormal cotyledon vein patterning

**Figure 4.**
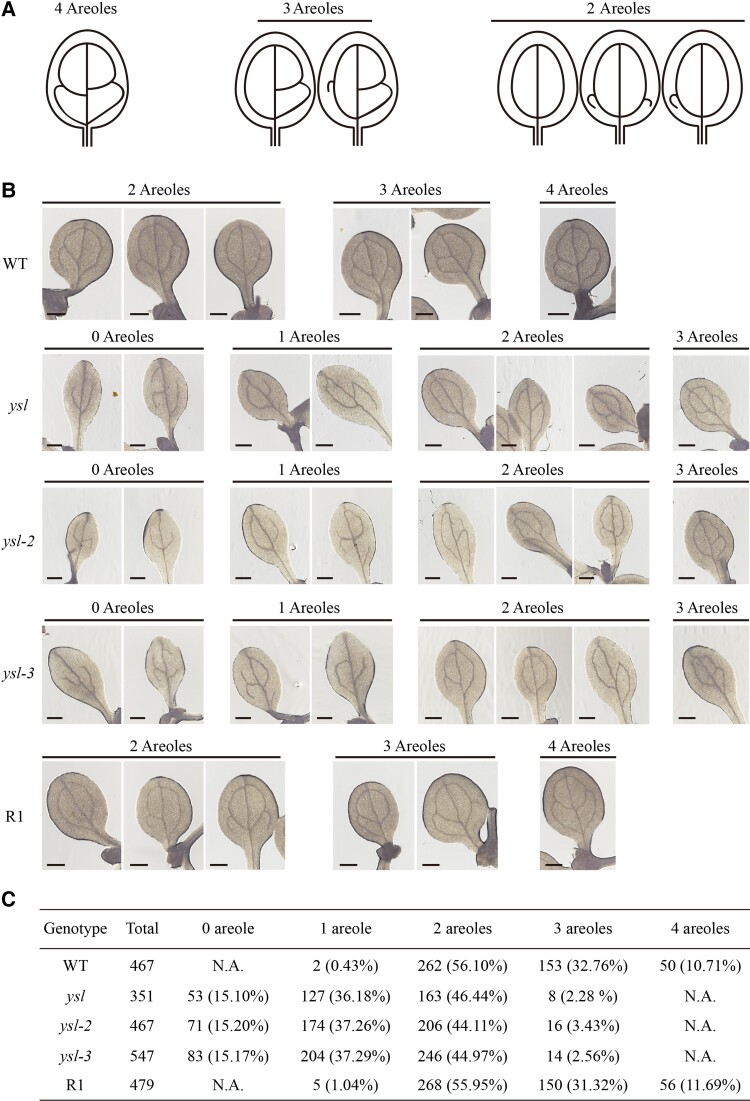
Cotyledon vein patterns in the wild type (WT), *ysl*, and rescued plants. **A)** Sketch of the cotyledon vein pattern in WT *Arabidopsis* plants. **B)** Representative 8-d-old cotyledon vein patterns with different numbers of areoles from WT, *ysl*, *ysl-2*, *ysl-3*, and rescued plants. Bars: 250 *μ*m. **C)** Quantification of areole numbers in cotyledons of the WT, *ysl*, *ysl-2*, *ysl-3*, and rescued plants. Cotyledons of 8-d-old WT, *ysl*, *ysl-2*, *ysl-3*, and rescued plants were used for this experiment. Percentages are indicated in parentheses. Total, cotyledon number; N.A., not applicable.

The serrated leaf phenotype of *ysl* and the activity of the *YSL* promoter in cotyledon veins prompted us to examine whether YSL functions in cotyledon vein patterning. In mature cotyledons, the number of closed areoles is an indicator of normal vein patterning (Sieburth 1999). In *Arabidopsis*, the predominant cotyledon venation pattern was 2, 3, or 4 areoles of normal vein patterning (Zheng et al. 2016) (Fig. 4A). We examined the cotyledon veins of 8-d-old WT, *ysl*, *ysl-2*, *ysl-3*, and rescued plants. In the *ysl*, *ysl-2*, and *ysl-3*, the secondary vein was disconnected in most cotyledons, which displayed the secondary vein did not link with the midvein or secondary vein did not connect each other (Fig. 4, B and C). In *ysl*, *ysl-2*, and *ysl-3*, many cotyledons had 0 areoles and 1 areole, and few had 3 areoles. However, in the WT and rescued plants, the cotyledon vein pattern was normal (Fig. 4, B and C). These results indicated that mutation of the *YSL* gene affects cotyledon vein patterning in *Arabidopsis*.

### YSL interacts with the vein developmental protein VIK

**Figure 5.**
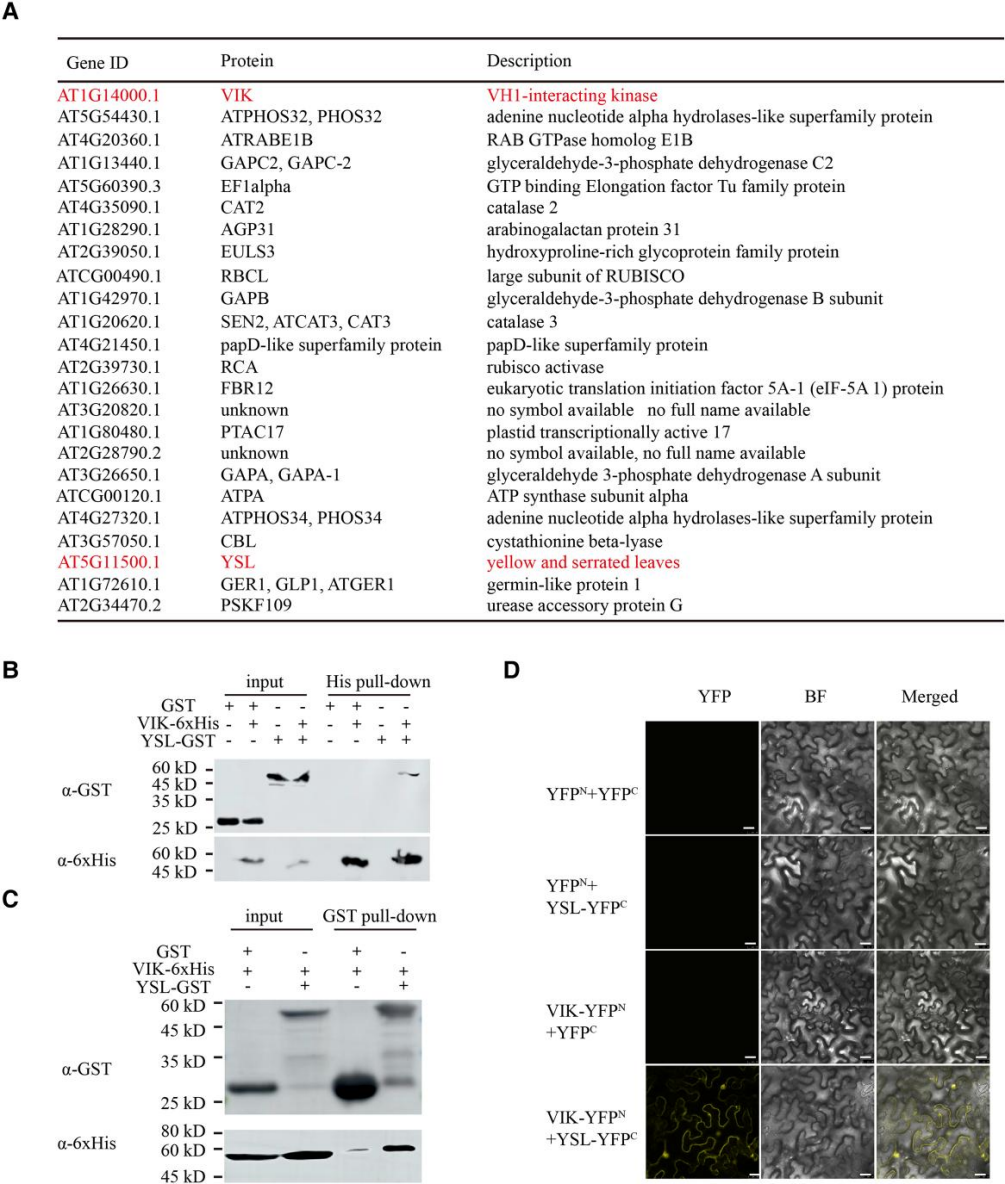
YSL interacts with VIK in *Arabidopsis*. **A)** Candidate YSL-interacting proteins identified by pull-down and mass spectrometry analyses. **B and C)** His/GST pull-down assay revealing the in vitro interaction between YSL and VIK. Protein samples were analyzed by immunoblotting using anti-His and anti-GST antibodies. +, add corresponding protein in the lane; −, do not add corresponding protein in the lane. **D)** Bimolecular fluorescence complementation (BIFC) assays showing the interaction of YSL with VIK. YFP fluorescence was observed when YSL-YFP^C^ was coexpressed with VIK-YFP^N^ in *N. benthamiana* leaves. Scale bars: 25 *μ*m.

The cotyledon vein patterning of *ysl* is defective. How does YSL function in *Arabidopsis* plants? Since YSL is a protein of unknown function in *Arabidopsis*, we hoped to identify YSL-interacting proteins. Therefore, we performed a pull-down assay to identify the interacting proteins. The YSL-His fusion protein was expressed in *Escherichia coli* and purified by His magnetic beads. Interacting proteins were pulled down from the total protein of *Arabidopsis* plants. We selected the 25 proteins with the highest abundance according to mass spectrometry analysis for further analysis (Fig. 5A). Interestingly, among these 25 proteins, the most abundant protein was VIK (a VH1-interacting kinase) (Fig. 5A), which was reported to be a vascular-specific protein that influences cotyledon vein pattern (Ceserani et al. 2009). To confirm the interaction, His/GST pull-down assays were subsequently performed in vitro, and bimolecular fluorescence complementation (BiFC) assays were subsequently performed in vivo. The results showed that both VIK and YSL could pull down each other, indicating that they interact in vitro (Fig. 5, B and C). The YFP signals of YSL-YFP^C^ and VIK-YFP^N^ were located in the nucleus and cell membrane, suggesting that YSL interacts with VIK in vivo (Fig. 5D). Since VIK is related to cotyledon vein development, the YSL protein may play an essential role in cotyledon vein development by interacting with VIK.

### *Ysl* displayed a reduced response to auxin in *Arabidopsis* leaves

**Figure 6.**
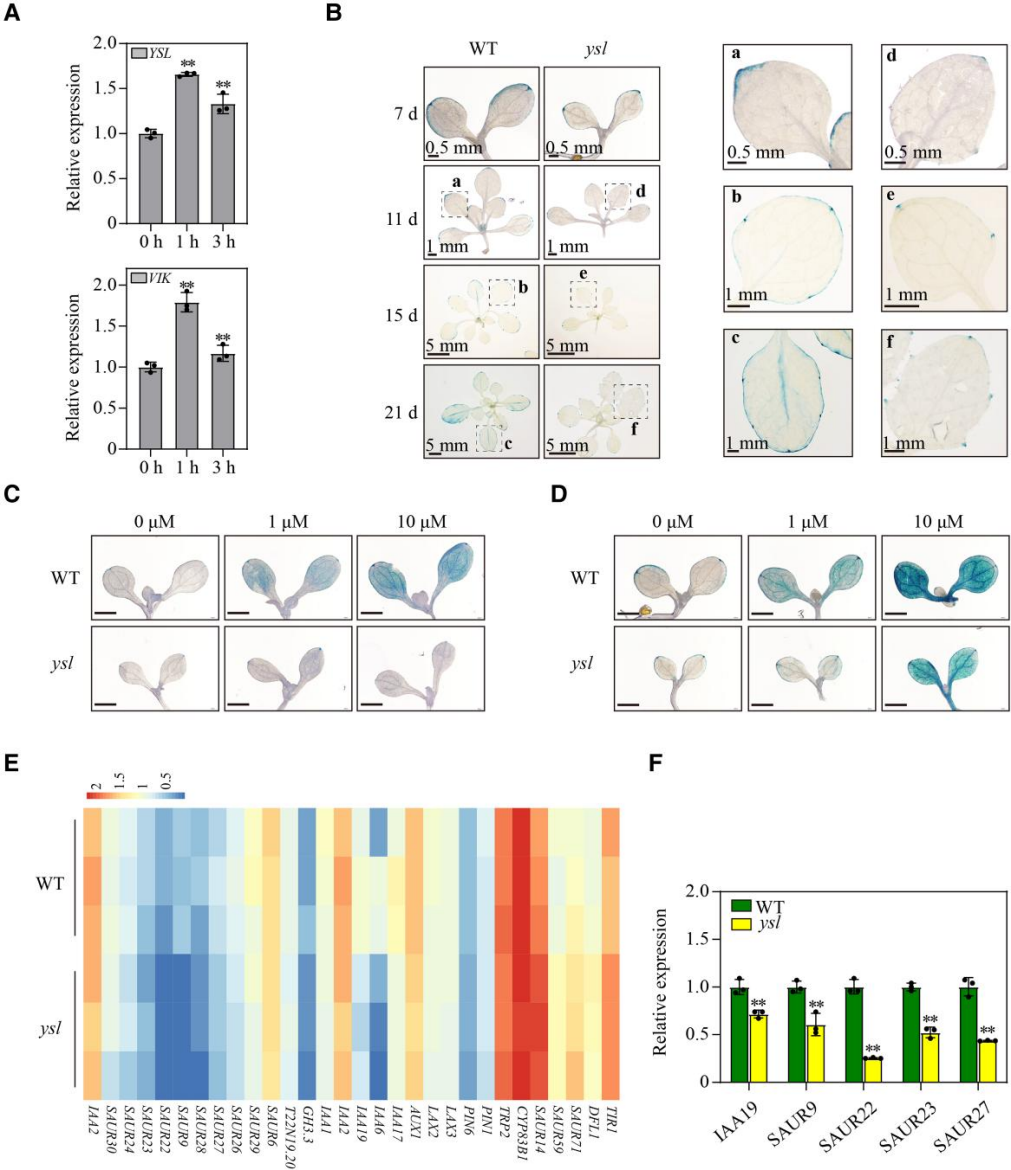
YSL affects the plant's transcriptional response to auxin and the expression of auxin-related genes. **A)** Relative expression of *YSL* and *VIK* in the wild type (WT) under IAA treatment. Eight-day-old WT plants were treated with 20 *μ*m IAA for 1 or 3 h. Values indicate means ± SD (*n* = 3). **Student's *t*-test with *P* < 0.01. **B)***DR5::GUS* expression pattern in the leaves of 7-, 11-, 15-, 21-d-old WT, and *ysl* plants. The a to f sections circled in the photographs are magnified and shown on the right. Three biological replicates were performed. **C** and **D)***DR5::GUS* expression pattern in cotyledons of 7-d-old WT and *ysl* plants treated without or with IAA. The plants were exposed to 0 *μ*m, 1 *μ*m, or 10 *μ*m IAA for 6 h. GUS staining for 3 to 4 h **C)** and 8 to 10 h **D)**. Bars, 1.5 mm. Three biological replicates were performed. **E)** Heatmap of the mRNA expression of auxin-related genes in WT and *ysl* plants. The 24-d-old WT and *ysl* seedlings were used for RNA-seq analysis. **F)** The relative expression of auxin-related genes in 24-d-old WT and *ysl* leaves was examined via RT-qPCR. **Values indicate means ± SD (*n* = 3). Student's *t*-test with *P* < 0.01.

Auxin plays a significant role in the formation of leaf margins and vascular development. VIK is involved in the establishment of vein patterns in foliar organs and influences auxin signaling (Ceserani et al. 2009). We next explored whether the function of *YSL* is related to auxin. We treated 8-d-old WT plants with 20 *μ*m IAA and found that the RNA expression of *YSL* and *VIK* was upregulated after IAA treatment (Fig. 6A), indicating that both *YSL* and *VIK* respond to auxin. DR5 is a reporter of auxin-dependent transcriptional response, we monitored the expression pattern of *DR5::GUS* in the WT and *ysl*. GUS signals were detected in the leaf tips and margins of 7-d-old WT and *ysl* plants (Fig. 6B). GUS-stained areas were distributed in the leaf tips, leaf margins, and leaf veins of 11, 15-d-old WT plants. In *ysl*, GUS was expressed mainly in leaf tips and teeth (Fig. 6B). *DR5::GUS* expression was detected mainly in the leaves, leaf tips, leaf margins, teeth, and leaf veins of 21-d-old WT plants. Nevertheless, in 21-d-old *ysl* mutants, GUS was expressed mainly in leaf tips and teeth (Fig. 6B). The altered GUS-stained areas in the mutants may partially explain the leaf shape and leaf vein pattern. To further explore the role of *YSL* in the auxin response, we detected the expression pattern of *DR5::GUS* in WT and *ysl* mutant plants treated with auxin. In the cotyledons of the WT plants, the expression of *DR5::GUS* was induced by exogenous IAA, whereas the expression of *DR5::GUS* in the *ysl* mutant was less sensitive to auxin after GUS staining for 3 to 4 h (Fig. 6C). Since increasing the GUS staining time can increase the strength of the GUS signal, we increased the GUS staining time to 8 to 10 h. *ysl* was responsive to exogenous IAA, but its sensitivity was significantly lower than that of the WT (Fig. 6D). To investigate the effect of YSL on auxin-related genes, we examined the transcription profiles of 24-d-old WT and *ysl* plants using RNA-seq (Supplementary Fig. S3). We selected 130 auxin-related genes which include 5 auxin biosynthesis-related genes (CYP83B1, TRP2, TRP3, NIT3, and AAO1) (Bartel et al. 2001), 6 auxin transport-related genes (Friml 2003), and 119 genes in KEGG auxin signal transduction pathway for analysis. In the 130 selected genes, 37 genes having an FPKM < 1 in both WT and *ysl*. Among the remaining 93 genes, 30 genes showed changes in expression (Supplementary Table S3). Among the differentially expressed genes, auxin transport-related genes (*PIN1* and *PIN6*), auxin biosynthesis-related genes (*CYP83B1*, *TRP2*) were downregulated in *ysl*. The expression of several genes involved in the auxin signal transduction pathway, including genes encoding auxin-induced proteins, auxin-responsive factors, and auxin influx proteins, were changed (Fig. 6E, Supplementary Table S3). Five genes (*IAA19*, *SAUR9*, *SAUR22*, *SAUR23*, and *SAUR27*) were verified by RT-qPCR to confirm the accuracy of the RNA-seq data (Fig. 6F). These results supported that *ysl* displayed a reduced response to auxin and changed the expression of auxin-related genes.

## Discussion

Leaf vein pattern and leaf margin morphology in vascular plants are regulated by a complex network involving numerous transcription factors, genes, small RNAs, and hormones (Sieburth 1999; Nikovics et al. 2006; Byrne 2012; Ruonala et al. 2017; Biedron and Banasiak 2018). In previous studies, several genes were reported to function in cotyledon vein patterning in plants. Here, we discovered a mutant named *ysl* with defective cotyledon vein patterns in *Arabidopsis* and cloned the *YSL* gene, which has not been previously reported in plants. Mutation of *YSL* affected not only cotyledon vein pattern and leaf marginal morphology but also leaf color (Figs. 1A and 4B). We performed a His pull-down assay to identify YSL-interacting proteins and identified VIK as a partner (Fig. 5, B to D). VIK is a vascular-specific protein. Furthermore, *vik* exhibit a defect in cotyledon vein connection (Ceserani et al. 2009). VIK can interact with another vein pattern development-related protein, VIH1. *VH1* is a provascular/procambial cell-specific gene. Loss of VH1 results in premature leaf senescence and possible defects in vascular transport (Clay and Nelson 2002). VH1/BRL2 receptor-like kinase interacts with the vascular-specific adaptor proteins VIT and VIK to influence leaf venation (Ceserani et al. 2009). YSL may participate in leaf venation development by interacting with VIK.

The protein sequence alignment revealed that the homologous proteins of YSL in eukaryotes are conserved (Supplementary Fig. S2A). To date, only the homologous protein CCDC25 has been reported in humans. In humans, CCDC25 is a transmembrane protein that acts as a receptor for NET-DNA on the cell membrane of cancer cells and can sense extracellular DNA (Yang et al. 2020). Its N-terminus interacts with NET-DNA, and its C-terminus interacts with an integrin-linked kinase (ILK), which regulates the process of cell migration and proliferation and subsequently activates the ILK-β-Parvin pathway to increase the motility and invasiveness of cancer cells (Yang et al. 2020). The YSL and CCDC25 have 59.26% amino acid similarity. They all contain a DUF814 (domain of unknown function) domain and a coiled coil region (Supplementary Fig. S4). The ILK contains the ankyrin repeats (Yang et al. 2020). VIK also bears N-terminally located ankyrin repeats (Ceserani et al. 2009). The YSL–VIK interaction in *Arabidopsis* is similar to the interaction between CCDC25 and ILK in humans. This interaction may be conserved in eukaryotes.

Auxin is a multifunctional trigger for plant development (Vanneste and Friml 2009). Cotyledon patterning, vascular differentiation, and serrated leaf marginal morphology are related to auxin (Sieburth 1999; Bilsborough et al. 2011; Biedron and Banasiak 2018; Du et al. 2018). In this study, we found that *YSL* and *VIK* responded to auxin (Fig. 6A) and reduced response to auxin in mutants (Fig. 6, B and C). YSL may affect auxin homeostasis in *Arabidopsis*. The reduced sensitivity of *ysl* to IAA (Fig. 6, C and D) indicated that *YSL* may also influence auxin signaling. According to the RNA-seq analysis, the expression of some auxin-related genes changed (Fig. 6E). YSL affected auxin signal transduction. The defective cotyledon vein pattern and serrated leaves of the mutants may be related to the homeostasis and level of auxin.

The developing veins appear to serve as morphogenetic centers that organize many of the differentiating leaf cells. In maize and some C4 plants, from research on the differentiation of mesophyll cells and bundle sheaths, the location of leaf veins can be determined as the first step in the patterning of surrounding photosynthetic cell types (Langdale et al. 1988; Langdale et al. 1989; Nelson and Dengler 1992). *ysl* mutants not only have an abnormal cotyledon vein phenotype but also have yellow leaves. The yellow leaf color of *ysl* may be due to leaf vein defects. In *vh1*, the chloroplasts of mesophyll cells were fewer, flattened, and lost many granal. Loss of VH1 results in premature leaf senescence and defective vascular transport (Clay and Nelson 2002). A defect in the vascular transport of photosynthates or antisenescence signals may induce leaf senescence, including the observed chloroplastic defects (Clay and Nelson 2002). We speculate that vein defects in the *ysl* affect vascular transport. The yellow color may be caused by compromised vascular transport or abnormal vascular-mesophyll cell associations. Leaf vein pattern defects affect vascular bundle transport, resulting in chloroplastic defects and ultimately resulting in leaf yellowing.

Auxin plays an important role in vein patterning in *Arabidopsis* (Sieburth 1999; Biedron and Banasiak 2018). Canalized auxin flow assumes that the formation of a vascular pattern occurs along the auxin flow which “canalized” into narrow strands. Vascular elements form along these strands (Nelson and Dengler 1997; Merks et al. 2007). The initially broad expression of the VH1 gene throughout the leaf primordium is restricted to only procambial (PC) cells (Clay and Nelson 2002), a pattern consistent with the hypothesis of signal canalization. It can be speculated that VH1 interacts with auxin, auxin-binding proteins, or a secondary signal generated by auxin perception. The signal is transduced through the kinase, resulting in effects on PC cell differentiation (Clay and Nelson 2002). VIK may function as a scaffold that links VH1 kinase activity to downstream activity, probably through a MAPK cascade. VIK belongs to the C1 subgroup of MAP kinase kinase kinase (Ichimura et al. 2002). VIK interacts with YSL and may phosphorylate YSL. YSL participates in the VH1–VIK pathway to influence leaf venation.

The existence of a leaf marginal serration is valuable. Moreover, more teeth and a larger tooth area may be related to the adaptation of plants to cold and wet environments (Burnham et al. 2001; Greenwood 2005; Royer et al. 2005). The mechanism of leaf marginal tooth formation remains poorly understood. The development of teeth depends on the formation of auxin maxima at leaf tips (Hay et al. 2006). In this study, we found a reduced response to auxin in the *ysl* mutant (Fig. 6, B and C) and that PIN1 was downregulated in *ysl* (Fig. 6E). It is possible that a mutation in *YSL* disrupts the polar localization of PIN1 in leaf margins and disperses PIN1 to more leaf margin cells. More auxin maxima are generated in the leaf margin, which leads to increased serrations. Moreover, mutation in *YSL* may alter the balance of the PIN1/CUC2 feedback loop, which leads to increased serrations. These findings provide some clues for us to elucidate the regulatory mechanism of leaf margin development, but the molecular mechanism underlying the effect of YSL on leaf margin teeth needs further study.

## Materials and methods

### Plant materials and growth conditions

The Arabidopsis (*A. thaliana*) ecotypes Columbia (Col-0) and Landsberg erecta (Ler) were used as wild-type (WT) plants in this study. *ysl* (locus *AT5G11500*) is a spontaneous mutant with a Col-0 background. In addition to the rough mapping experiment in which we used Ler, the WT plants in this study represented Col-0 plants. *Arabidopsis* seeds were sterilized and subsequently planted on 1/2 MS media supplemented with 1% sucrose (w/v) and 0.8% (w/v) agar. After stratification at 4 °C in darkness for 2 d, the seeds were placed in a greenhouse at 22 °C with a 16 h light/8 h dark cycle. The light intensity was maintained at 80 *μ*mol photons m^−2^ s^−1^. Seven-day-old plants were transplanted to soil under the same conditions. The day-old plates were removed after the plants reached 4 °C for growth.

In this study, *N. benthamiana* seeds were sown on soil in a greenhouse at 25 °C with a 16 h light/8 h dark cycle. Five-week-old plants were subjected to transient expression.

### Chlorophyll and chlorophyll fluorescence measurements

The chlorophyll content of the rosette leaves of the Arabidopsis plants was measured. Chlorophyll was extracted with 80% (v/v) acetone and quantified on a spectrophotometer. We measured chlorophyll with a NanoDrop 2000c (Thermo Fisher) and calculated the chlorophyll content with the following formula: Chl a = 12.21 × A_663_ − 2.81 × A_645_; Chl b = 20.13 × A_645_ − 5.03 × A_663_.

The chlorophyll imaging system FluorCam FC 800-C/1010 (PSI) was used to measure chlorophyll fluorescence. The plants were kept in the dark for 20 min before testing. *F*_o_ represents the value of minimal fluorescence in the dark-adapted state. *F*_m_ represents the value of maximum fluorescence in the dark-adapted state. *F*_m_′ is the value of the maximum fluorescence value in the light-adapted state. The maximum efficiency of PSII photochemistry (*F*_v_/*F*_m_) was calculated as (*F*_m_−*F*_o_)/*F*_m_.

### Leaf margin analysis and cotyledon vein pattern observation

The fifth true leaves of 3-wk-old plants were photographed using a stereomicroscope (SMZ25, Nikon, Japan) with a CCD camera. Three parameters were used in the leaf margin analysis: the leaf dissection index (perimeter^2^/4π × leaf area), the number of teeth/leaf perimeter, and the tooth area/leaf area (Royer et al. 2008; Bilsborough et al. 2011). Leaf area and perimeter were measured with NIS-Elements (Nikon) software.

Cotyledons of 8-d-old plants (from the WT, *ysl*, and rescued lines) were selected for observing the vein pattern. The cotyledons were decolorized with 75% ethanol (v/v) and 25% acetic acid (v/v) until all chlorophyll decomposition. Veins were observed with a stereomicroscope (SMZ25, Nikon, Japan).

### Positional cloning of *YSL*

We used rough mapping and BSA to screen for mutant genes. *ysl* (Col background) was crossed with Ler to generate the F_1_ population. Eighty-eight individuals of the F_2_ population with a mutant phenotype were subjected to rough mapping. The marker primers used were selected from the Arabidopsis mapping platform (AMP) (Hou et al. 2010), and 25 SSLP markers (Supplementary Table S4) were used for rough mapping.

*ysl* (Col-0 background) was crossed with Col-0 to generate the F_2_ sequencing population. DNA was extracted from 50 individual plants from the 2 pools, after which a library was constructed. The DNA of the WT plants was pooled into 1 pool, and the DNA of the mutant plants was pooled into another pool. These 2 pools were used for BSA. HiSeq. A 4000 system (Illumina) was used for high-throughput sequencing. Clean data were used for analyzing single nucleotide polymorphisms (SNPs) and insertion–deletions (InDels). The genome sequences of the 2 pools were aligned with the *Arabidopsis* reference genome. The screening criterion was a SNP index ≥ 0.9.

### Plant transformation, rescue analysis, and construction of knockout plants

To rescue the mutant phenotype, the CDS of *YSL* (*AT5G11500*) was amplified from WT cDNA using the primers YSLrescue-F and YSLrescue-R, as shown in Supplementary Table S1. The PCR product was cloned and inserted into the donor vector pDONR223 by a BP clonase reaction via the Gateway site. After that, the attL recombination clonase (Invitrogen) reaction was performed to introduce the fusion construct into the destination vector pEarleyGate101 (35S promoter, YFP, and HA tags). Subsequently, the constructs were subsequently transformed into *ysl* mutants via the floral-dip method (Clough and Bent 1998). The seeds were screened on 1/2 MS media supplemented with the 7 *μ*g/ml herbicide Basta. The transgenic plants were selected for the next experiment.

We used the pRGEB35 vector to construct a knockout vector via CRISPR-Cas9 technology according to a method described previously (Xie et al. 2015). The constructs were subsequently transferred into WT plants. Transgenic plants were screened on 1/2 MS media supplemented with 50 mg/ml hygromycin. The primers used (YSLcispr-1F, YSLcispr-1R, YSLcispr-2F, and YSLcispr-2R) for constructing the CRISPR vector are listed in Supplementary Table S5.

### Protein extraction and immunoblot analysis

Total protein was extracted from the WT, *ysl* mutant, and rescued plants with total protein extraction buffer containing 50 mm Tris-HCl (pH = 7.5), 150 mm NaCl, 0.5% Triton X-100, and protease inhibitor cocktail. The protein samples were separated by 12% SDS–PAGE and subsequently transferred to nitrocellulose membranes. The membranes were incubated with an HA antibody (ABclonal, AE005) and SuperSignal West Pico PLUS Chemiluminescent Substrate (Thermo Scientific) to detect the signals.

### GUS staining analysis

To examine the tissue-specific expression patterns of *YSL*, we cloned a 2 kb fragment before the start codon of *AT5G11500* into a vector with a GUS reporter. The *YSLPro::GUS* construct in *Agrobacterium tumefaciens* strain GV3101 was subsequently introduced into WT plants. The *YSLPro::GUS* transformants were subjected to GUS staining analysis. For GUS staining, we used 50 mm sodium phosphate buffer containing 1 mg/ml X-Gluc, 10 mm EDTA, 1% (v/v) Triton X-100, 2 mm K_3_[Fe(CN)_6_], and 2 mm K_4_[Fe(CN)_6_]. The GUS-stained plants were soaked in 75% ethanol, after which the chlorophyll was removed with 75% ethanol and 25% acetic acid. The samples were observed under a stereomicroscope (SMZ25, Nikon, Japan) and photographed using a digital camera. Three biological replicates were performed. For the analysis of *DR5::GUS* expression in *ysl* mutants, the *ysl* mutants were crossed with *DR5::GUS* transgenic plants. To analyze the auxin response, cotyledons of 1-wk-old plants were treated with 0 *μ*m, 1 *μ*m, or 10 *μ*m IAA for 6 h and then subjected to subsequent histochemical detection. Three biological replicates were performed.

### RNA extraction, reverse transcription PCR, and RT-qPCR analysis

Total RNA was isolated from tissue samples with TRIzol (TransGen). cDNA was synthesized using the PrimeScript RT Reagent Kit (Takara). qPCR analysis was performed to detect the gene expression levels. qPCR amplification was carried out in a 7300 Plus RT-qPCR system (ABI) using TB Green Premix Ex Taq II (Tli RNaseH Plus; Takara). The *actin2* gene was used as an endogenous control. The relative expression was calculated using the formula 2^−ΔΔ^C_t_. The experiment was performed with 3 technical replicates. The primers used for RT-qPCR are listed in Supplementary Table S5.

### Subcellular localization

The CDS of *YSL* was cloned and inserted into the pEarleyGate101 vector, which expresses the YSL-YFP-HA fusion protein under the control of the 35S promoter. The mutants were stably transformed with the pEarley101-YSL-YFP transformants via the floral dipping method. Transgenic plants were confirmed by Basta selection, and homozygous lines were used for the experiment. The rosette leaves, roots, and protoplasts of the rosette leaves of the homozygous rescued plants were used for subcellular localization. *Arabidopsis* protoplasts were isolated as previously described (Yoo et al. 2007). Rosetta leaves of 3-wk-old *Arabidopsis* plants were cut into slices and digested with Cellulase R-10 and Macerozyme R-10. The protoplasts were filtered, washed, and resuspended in MMG solution. In the subcellular localization experiment of *N. benthamiana* transient expression, GV3101 cells containing 35S:YSL-YFP, 35S:H2A. Z-CFP, and 35S:PIP2A-mcherry were expressed in the leaves of *N. benthamiana*. H2A. Z and PIP2A were used for the nuclear marker and cell cell membrane marker (González et al. 2005; Kumar 2018). The YFP signals were visualized using a confocal laser-scanning microscope (TCSSP8, Leica Microsystems, Germany). The YFP signal excitation was at 514 nm, and the emission signals were collected between 520 and 550 nm. The CFP signal excitation was at 405 nm, and the emission signals were collected between 450 and 480 nm. The mcherry signal excitation was at 552 nm, and the emission signal was collected between 600 and 630 nm. Three biological replicates were performed.

### GST/His pull-down and BiFC assays

The CDSs of the corresponding genes were cloned and inserted into expression vectors with GST or His tags. The expression vectors were subsequently transformed into *E. coli* Rosetta, after which the proteins were expressed at 16 °C. For the pull-down assay, the expressed proteins were incubated with BeaverBeads IDA-Nickel (Beaver Biosciences, China) at 4 °C for 4 h. The bound proteins were separated via SDS–PAGE and detected via His (ABclonal, AE003) and GST (PhytoAB, PHY5013) antibodies.

For the BiFC assay, the target proteins were fused to the N- or C-terminal region of YFP and coexpressed in *N. benthamiana* leaves using agroinfiltration. The fluorescent signals were evaluated with a confocal microscope (TCSSP8, Leica Microsystems, Germany) after 36 to 48 h. The YFP signal excitation was at 514 nm, and the emission signals were collected between 520 and 550 nm. Three biological replicates were performed. The primers used for constructing the expression vector are listed in Supplementary Table S5.

### RNA-seq analysis

Total RNA was extracted from the leaves of the WT and *ysl* plants at the 24-d-old stage. mRNA was enriched from total RNA and then fragmented. Random hexamer primers were used for reverse transcription. The library was prepared and sequenced using the Illumina HiSeq X platform. Clean reads were aligned to the reference genome using HISAT2 v2.0.4. The expected number of fragments per kilobase of transcript sequence per million base pairs sequenced (FPKM) of each gene was calculated based on the length of the gene and the read count mapped to that gene.

### Phylogenetic analyses

MEGA software (MEGA 5.1) and the Neighbor-Joining (NJ) method were used to construct phylogenetic tree. A total of 1,000 bootstrap analyses ensure statistical support for topology. The steps were as follows: first, calculating genetic distance matrices based on the evolutionary model of the sequences; secondly, constructing an initial phylogenetic tree with the Neighbor-Joining method. Then, 1,000 bootstrap resamplings were conducted, with a new phylogenetic tree reconstructed after each resampling. Finally, bootstrap support values were listed on the initial tree.

### Statistical analyses

Statistical analysis of the results of the bioassays was performed with the DPS software package. Data represent the means ± SD from at least 3 replicates; different uppercase letters above the bars indicate significant differences among means (*P < 0.05; **P < 0.01), as calculated with Student's *t*-test.

### Accession numbers

The sequence information for this study can be found in The Arabidopsis Information Resource (TAIR) under the following accession numbers: *YSL* (*AT5G11500*), *VIK* (*AT1G14000*), *IAA19* (*AT3G15540*), *SAUR9* (*AT4G36110*), *SAUR22* (*AT5G18050*), *SAUR23* (*AT5G18060*), and *SAUR 27* (*AT3G03840*).

## Supplementary Material

kiae465_Supplementary_Data

## Data Availability

Data supporting the findings of this work are available within the article and its Supporting Information files. The RNA-seq raw data have been deposited in the CNCB database with BioProject number PRJCA027855.

## Supplementary data

The following materials are available in the online version of this article.

**Supplementary Figure S1.** Rough mapping of the *YSL* gene.

**Supplementary Figure S2.** Sequence analysis of YSL.

**Supplementary Figure S3.** RNA-seq analysis of 24-d-old WT and *ysl* rosette leaves in soil.

**Supplementary Figure S4.** Sequence analysis of YSL and CCDC25.

**Supplementary Table S1.** Crossover ratio of rough mapping.

**Supplementary Table S2.** Indels and SNPs in candidate genes.

**Supplementary Table S3.** Expression of auxin-related genes in RNA-seq analysis.

**Supplementary Table S4.** SSLP makers for rough mapping.

**Supplementary Table S5.** Primers used for vector construct or qPCR.

## Dive Curated Terms

The following phenotypic, genotypic, and functional terms are of significance to the work described in this paper:

PIN1 Gramene: AT1G73590
PIN1 Araport: AT1G73590

NIT3 Gramene: AT3G44320
NIT3 Araport: AT3G44320

AAO1 Gramene: AT5G20960
AAO1 Araport: AT5G20960

BRL2 Gramene: AT2G01950
BRL2 Araport: AT2G01950

At5g11500 Gramene: AT5G11500
At5g11500 Araport: AT5G11500

CVP2 Gramene: AT1G05470
CVP2 Araport: AT1G05470

CYP83B1 Gramene: AT4G31500
CYP83B1 Araport: AT4G31500

PIN6 Gramene: AT1G77110
PIN6 Araport: AT1G77110

SFC Gramene: AT5G13300
SFC Araport: AT5G13300

VIK Gramene: AT1G14000
VIK Araport: AT1G14000

auxin CHEBI: CHEBI:22676

HISAT2 Gramene: High stacks
HISAT2 Araport: High stacks

KEGG Gramene: Kyoto Encyclopedia of Genes and Genomes
KEGG Araport: Kyoto Encyclopedia of Genes and Genomes
