# Supplementary material for: YELLOW, SERRATED LEAF is essential for cotyledon vein patterning in Arabidopsis
Source: Plant Physiol. 2024 Sep 3;196(4):2504–16. doi: 10.1093/plphys/kiae465 (PMC11637768; doi:10.1093/plphys/kiae465)
Supplement: kiae465_Supplementary_Data [file kiae465_supplementary_data.zip › PP2024RA00256R2_Supplemental_Figure_1.pdf]

Supplementary Figure S1

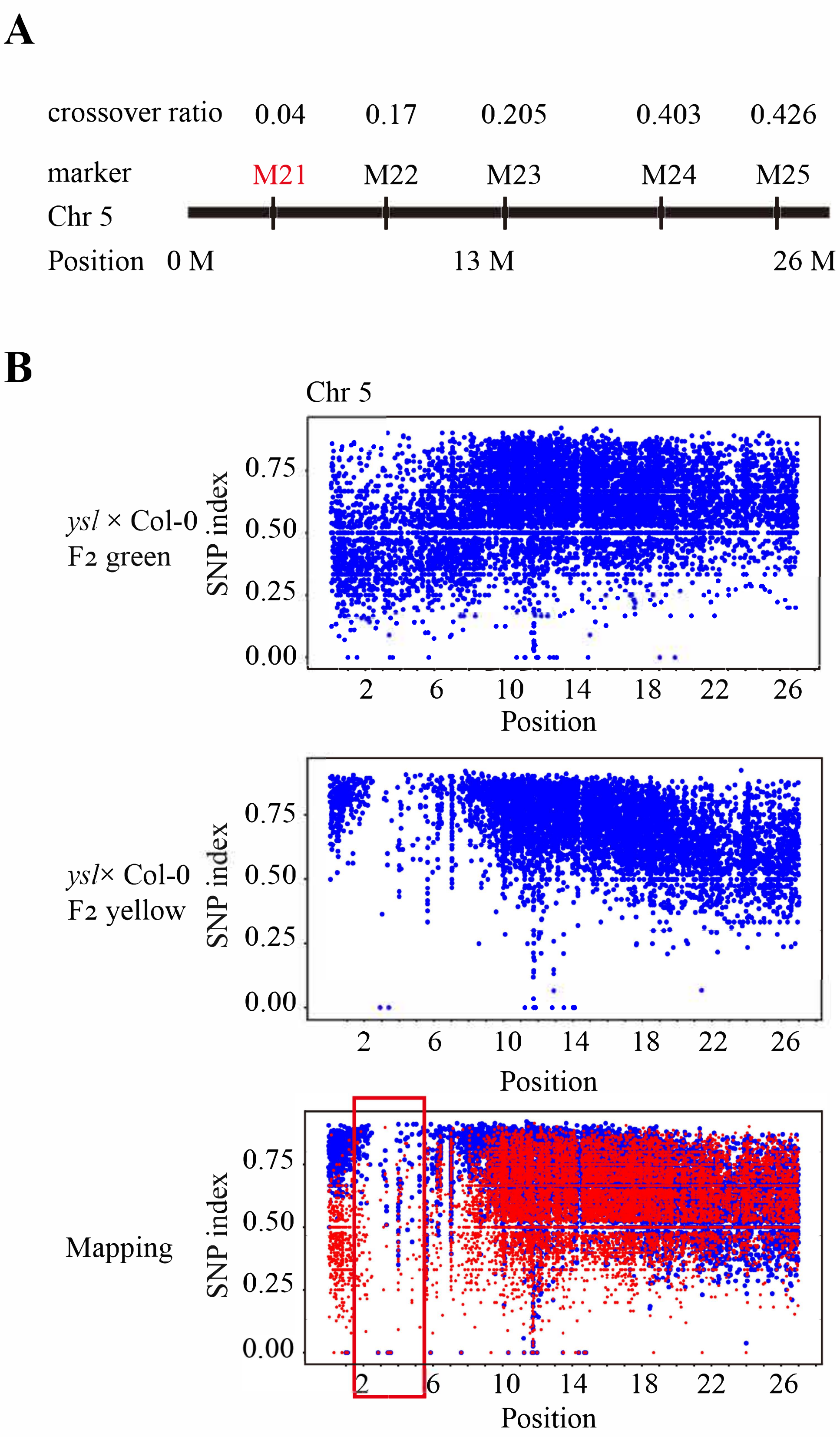

Figure S1. Rough mapping of the YSL gene. **A)** Chromosome rough mapping of the *YSL* gene. M21, M22, M23, M24 and M25 represent the SSLP markers on chromosome 5. The crossover ratio was used to determine the mutant region. **B)** Bulked segregant analysis (BSA) of *ysl* mutants. The red box represents the mutant candidate region. The blue and red dots represent the numerical value of SNP index. The blue and red dots represent the numerical value of single nucleotide polymorphism (SNP) index.

Supplementary Figure S2

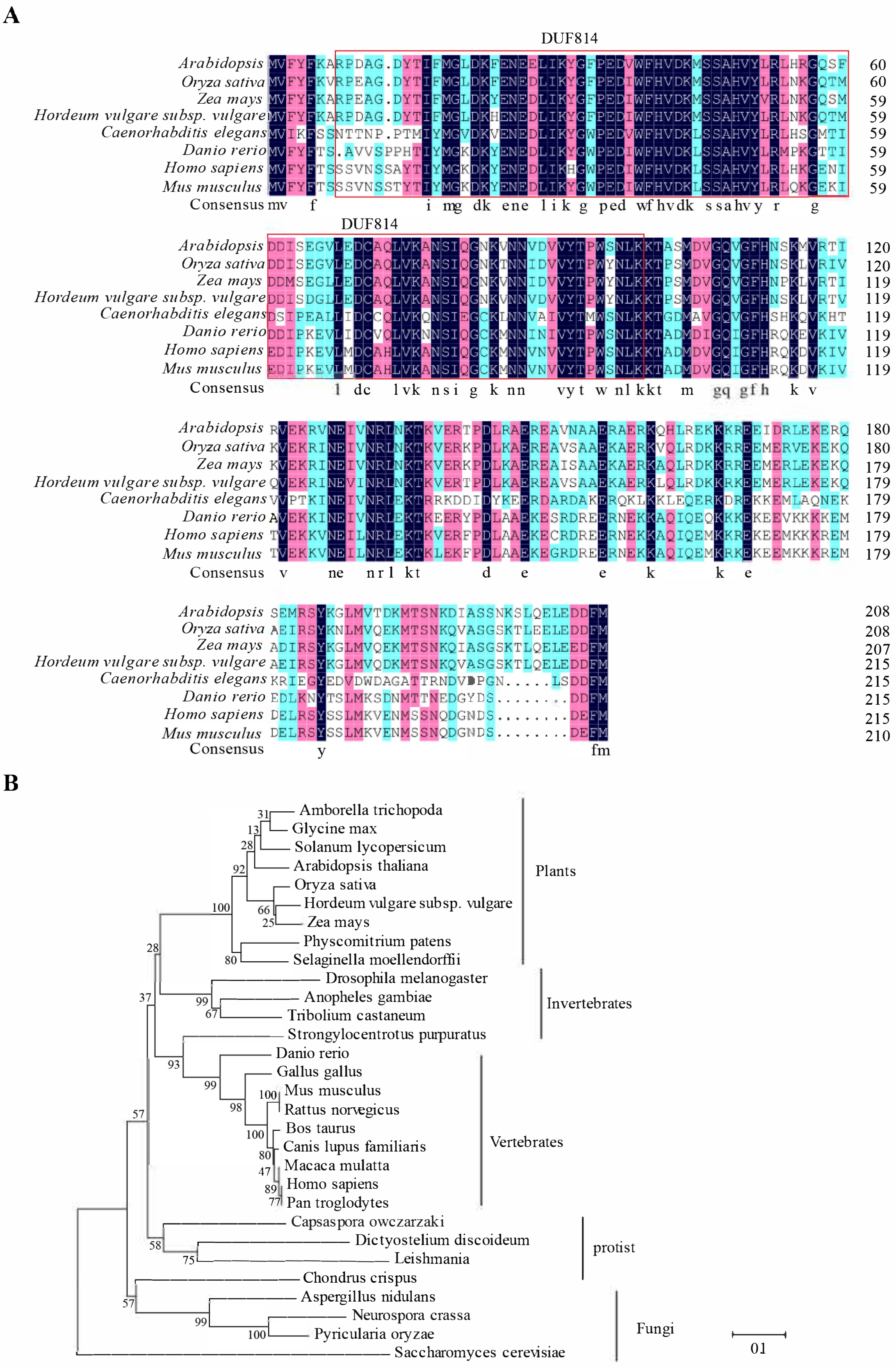

Figure S2. Sequence analysis of YSL. A) Multiple sequence alignment of homologous YSL proteins in different species. The red frame represents the conserved DUF814 (domain of unknown function) domain. **B)** Phylogenetic tree of YSL members from different species. The accession numbers are as follows: *Saccharomyces cerevisiae S288C*, NP_013851.1; *Pyricularia oryzae*, XP_00370931 8.1; *Neuros-pora crassa OR74A*, XP_963522.1; *Aspergillus nidulans FGSC A4*, XP_050467454.1; *Chondrus crispus*, XP_005713749.1; *Leishmania major strain Friedlin*, XP_001684812.1; *Dictyostelium discoideum AX4*, XP_646276.1; *Capsaspora owczarzaki ATCC 30864*, XP_004349419.2; *Caenorhabditis elegans*, NP_496971.1; *Strongylocentrotus purpuratus*, XP_030838868.1; *Tribolium castaneum*, XP_971943.2; *Apis mellifera*, XP_006572231.1; *Anopheles gambiae str. PEST*, XP_313381.4; *Drosophila melanogaster*, NP_001284975.1; *Homo sapiens*, NP_06071 6.2; *Pan troglodytes*, XP_519679.3; *Macaca mulatta*, NP_001253501.1; *Mus musculus*, NP_666056.1; *Rattus norvegicus*, NP_001101852.1; *Gallus gallus*, XP_040524310.1; *Bos taurus*, NP_001030216.1; *Canis lupus familiaris*, XP_038291268.1; *Danio rerio*, NP_956682.1; *Amborella trichopoda*, XP_006833314.1; *Glycine max*, NP_001241216.1; *Solanum lycopersicum*, XP_004245263.1; *Arabidopsis thaliana*, NP_196711.1; *Oryza sativa Japonica Group*, XP_015640648.1; *Hordeum vulgare subsp. vulgare*, XP_044967884.1; *Zea mays*, NP_001150110.3; *Physcomitrium patens*, XP_024384515.1; *Selaginella moellendorffii*, XP_002992576.1.

Supplementary Figure S3

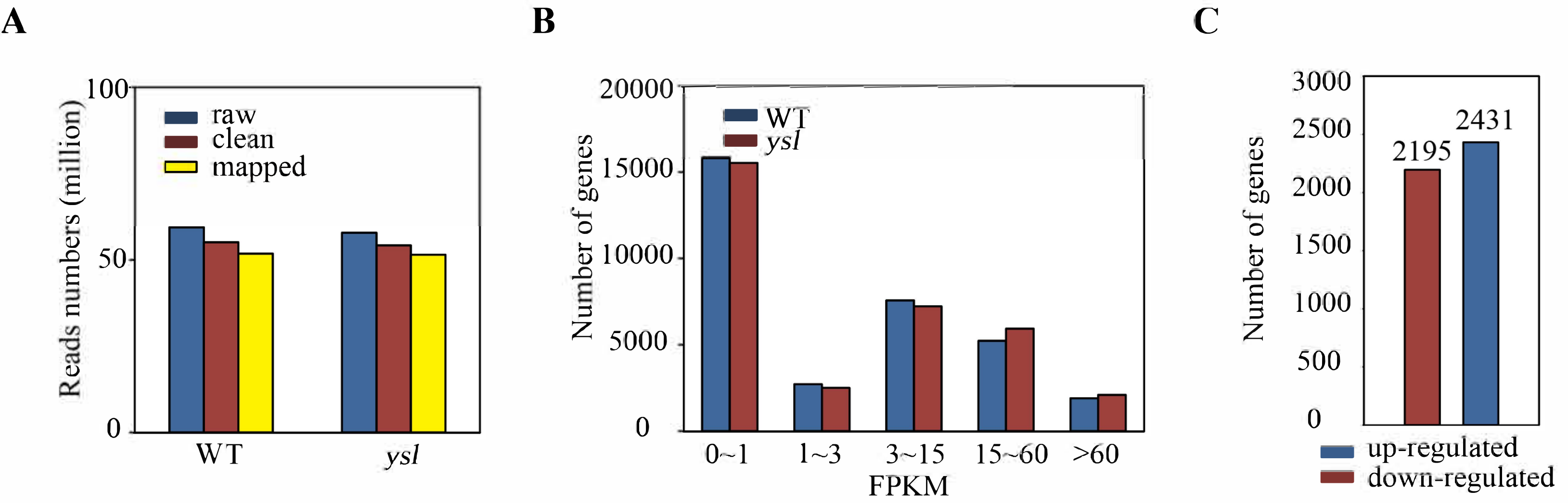

Figure S3. RNA-seq analysis of 24-day-old wild type (WT) and *ysl* rosette leaves in soil. A) Read numbers of sample sequences. **B)** Frequencies of the detected genes sorted according to expression level. FPKM, Fragments Per Kilobase Million. **C)** Number of differentially expressed transcripts.

SupplementaryFigure S4

A

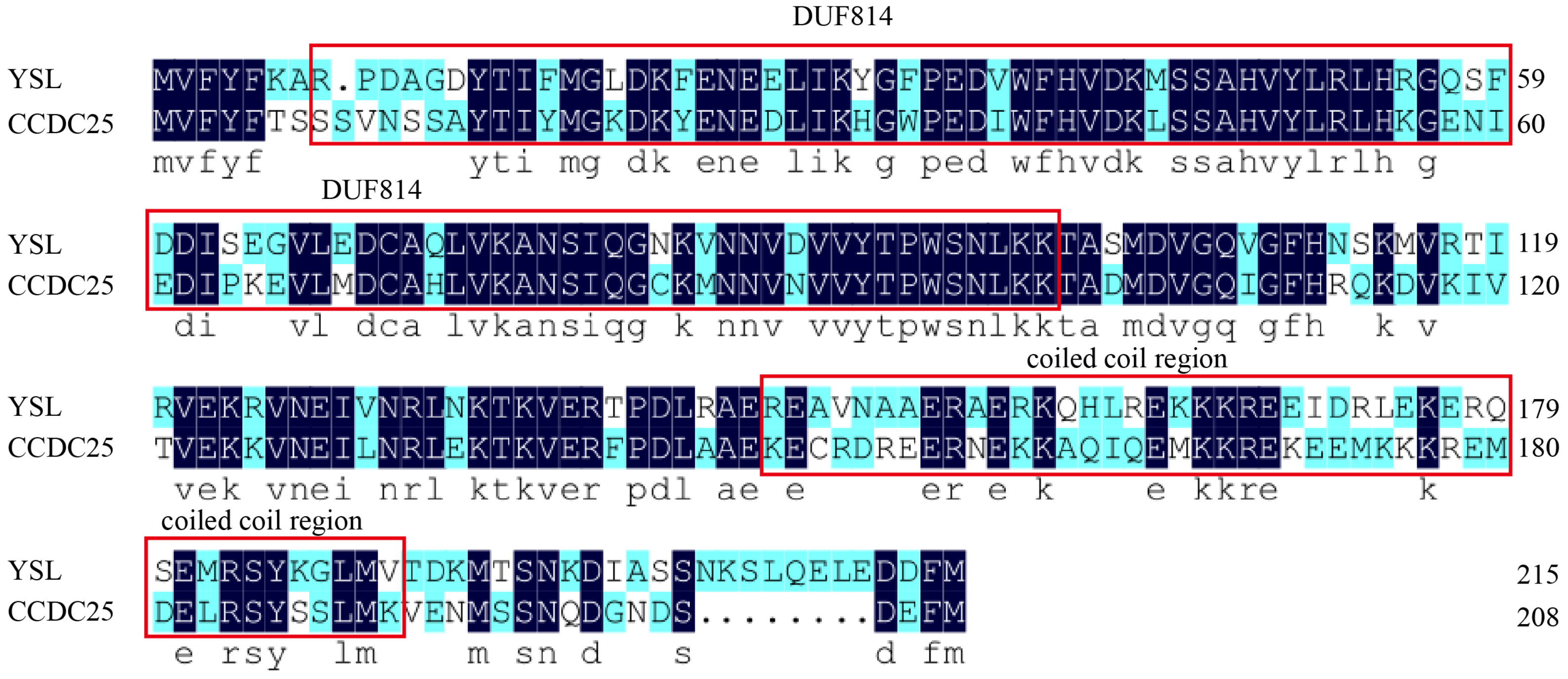

B

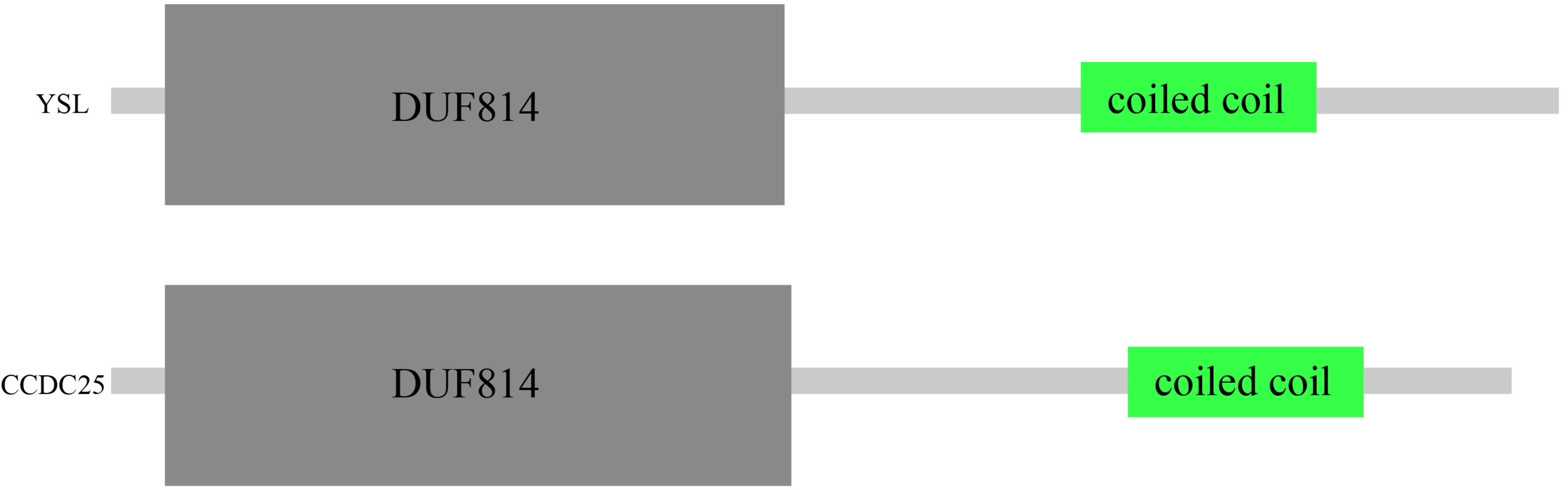

Figure S4. Sequence analysis of YSL and CCDC25. **A)** The amino acid sequence alignment of YSL and CCDC25. Red boxes represent the conserved DUF814 domain and the coiled coil region. **B)** The conserved domain of YSL and CCDC25.
