# Supplementary material for: YELLOW, SERRATED LEAF is essential for cotyledon vein patterning in Arabidopsis
Source: Plant Physiol. 2024 Sep 3;196(4):2504–16. doi: 10.1093/plphys/kiae465 (PMC11637768; doi:10.1093/plphys/kiae465)
Supplement: kiae465_Supplementary_Data [file kiae465_supplementary_data.zip › PP2024RA00256R2_Supplemental_Table_1_5.pdf]

Supplementary Table S1. Crossover ratio of rough mapping.

Maker in chromosome 1	Single crossing over	Double crossing over	Crossing-over ratio
M1	40	23	0.489
M2	45	20	0.483
M3	46	19	0.477
M4	48	18	0.477
M5	49	16	0.460

Maker in chromosome 2	Single crossing over	Double crossing over	Crossing-over ratio
M6	35	15	0.369
M7	25	13	0.290
M8	46	14	0.420
M9	53	11	0.426
M10	47	27	0.574

Maker in chromosome 3	Single crossing over	Double crossing over	Crossing-over ratio
M11	39	28	0.540
M12	44	26	0.545
M13	51	25	0.574
M14	46	26	0.557
M15	46	18	0.466

Maker in chromosome 4	Single crossing over	Double crossing over	Crossing-over ratio
M16	44	23	0.511
M17	47	22	0.517
M18	50	21	0.523
M19	43	23	0.506
M20	41	26	0.528

Maker in chromosome 5	Single crossing over	Double crossing over	Crossing-over ratio
M21	5	1	0.040
M22	0	15	0.170
M23	28	4	0.205
M24	49	11	0.403
M25	47	14	0.426

Supplementary Table S2. Indels and SNPs in candidate genes.

Position	Reference	Alteration	SNP_index	Mutation site or type	Gene
2679052	ACC	AC	1	upstream	AT5G08310
3394560	GAAA	GAAAA	1	upstream	AT5G10730
3406993	AGG	AG	0.94	upstream	AT5G10760
3481429	T	TA	1	upstream	AT5G11000
3491263	CA	CAA	1	3'UTR	AT5G11030
3530431	AT	ATT	1	upstream	AT5G11100
3566883	TCCCC	TCCC	1	upstream	AT5G01855
3576889	CTTTTT	CTTTT	1	upstream	AT5G11210
3620753	ATT	ATTT	1	upstream	AT5G11330
3620790	CTTT	CTTTT	1	upstream	AT5G11330
3676758	CTT	CTTT	1	frameshift mutation	AT5G11500
3694351	GA	GAA	1	upstream	AT5G11530
4622758	AT	ATT	1	upstream	AT5G14330
4879508	CG	CGG	1	intron	AT5G15070
5460050	CAAAA	CAAAAAA	1	upstream	AT5G16640
5460053	AA	AAAATA	1	upstream	AT5G16640
5485195	AT	ATT	1	upstream	AT5G16715
2821040	G	T	1	missense mutation	AT5G08670
2848835	T	C	1	missense mutation	AT5G08740
2848840	A	T	1	missense mutation	AT5G08740
2906922	A	T	1	missense mutation	AT5G09360
2906926	T	C	1	missense mutation	AT5G09360
2906934	C	A	1	missense mutation	AT5G09360
2906940	G	T	1	missense mutation	AT5G09360
3406839	T	C	1	upstream	AT5G10760
3406841	T	C	1	upstream	AT5G10760
3406891	T	C	1	upstream	AT5G10760
3406894	T	C	1	upstream	AT5G10760
3406903	G	T	1	upstream	AT5G10760
3406917	G	T	1	upstream	AT5G10760
3406933	T	C	1	upstream	AT5G10760
3437528	C	G	1	missense	AT5G10900
3437530	C	G	1	missense	AT5G10900
3506582	A	C	1	upstream	AT5G11060
3506584	A	C	1	upstream	AT5G11060
3506586	A	G	1	upstream	AT5G11060
3506659	G	C	1	upstream	AT5G11060
3506660	A	T	1	upstream	AT5G11060
3576885	T	C	1	upstream	AT5G11210
3576920	A	T	1	upstream	AT5G11210
3576921	G	A	1	upstream	AT5G11210
3576922	C	G	1	upstream	AT5G11210

Supplementary Table S3. Expression of auxin-related genes in RNA-seq analysis.

FPKM_ WT	FPKM_ *ysl*	Log2 Fold Change	Padj	Regulation	Gene	Gene name
162.86	153.76	-0.29901	0.01389	DOWN	AT4G31500	CYP83B1
86.99	85.02	-0.24957	0.03851	DOWN	AT5G54810	TRP2
5.84	4.84	-0.48881	0.00765	DOWN	AT1G73590	PIN1
2.22	1.60	-0.68726	0.01150	DOWN	AT1G77110	PIN6
9.15	7.85	-0.43711	0.00498	DOWN	AT1G77690	LAX3
10.28	8.19	-0.54883	0.00704	DOWN	AT2G21050	LAX2
39.55	33.26	-0.46693	2.52E-05	DOWN	AT2G38120	AUX1
14.42	8.77	-0.92997	0.01581	DOWN	AT1G04250	IAA17
5.68	0.955	-2.7839	0.03817	DOWN	AT1G52830	IAA6
9.97	4.65	-1.3122	3.05E-05	DOWN	AT3G15540	IAA19
49.63	41.86	-0.46095	0.00713	DOWN	AT3G23030	IAA2
10.88	7.88	-0.68511	0.01093	DOWN	AT4G14560	IAA1
1.58	0.94	-0.96059	0.00343	DOWN	AT2G23170	GH3.3
7.76	6.84	-0.40018	0.02029	DOWN	AT5G13370	T22N19.20
32.37	29.01	-0.37809	0.03609	DOWN	AT2G21210	F26H11.3
12.14	8.33	-0.76199	0.01345	DOWN	AT3G03820	SAUR29
6.56	4.47	-0.77034	0.00552	DOWN	AT3G03850	SAUR26
4.54	2.59	-1.0252	0.00206	DOWN	AT3G03840	SAUR27
1.98	0.697	-1.7328	0.01848	DOWN	AT3G03830	SAUR28
2.51	0.459	-2.6674	1.23E-09	DOWN	AT4G36110	SAUR9
1.44	0.382	-2.1355	0.00136	DOWN	AT5G18050	SAUR22
3.03	1.68	-1.0663	0.00883	DOWN	AT5G18060	SAUR23
6.12	3.91	-0.86659	0.00568	DOWN	AT5G18080	SAUR24
8.32	6.40	-0.59606	0.00628	DOWN	AT5G53590	MNC6.13
49.63	41.86	-0.46095	0.00713	DOWN	AT3G23030	IAA2
44.79	69.06	0.40454	0.04492	UP	AT3G62980	TIR1
9.17	15.40	0.52992	0.00461	UP	AT5G54510	DFL1
13.91	31.56	0.96844	0.00287	UP	AT1G56150	F14G9.23
9.24	15.42	0.52051	0.00195	UP	AT3G60690	SAUR59
68.38	113.24	0.51108	2.05E-05	UP	AT4G38840	F19H22.7
6.58	6.39	-0.25443	0.6932	—	AT3G44320	NIT3
2.67	3.99	0.36846	0.05220	—	AT5G20960	AAO1
50.48	51.01	-0.20233	0.16359	—	AT3G54640	TRP3
0.0605	0.0743	0.075856	1	—	AT5G57090	EIR1
42.66	42.14	-0.23411	0.11745	—	AT1G70940	PIN3
48.99	59.25	0.05849	0.84317	—	AT2G01420	PIN4
40.26	43.74	-0.097966	0.57721	—	AT1G23080	PIN7
41.46	49.13	0.029858	0.99532	—	AT5G01240	LAX1
1.03	1.65	0.45993	0.40966	—	AT1G04100	IAA10
20.97	10.72	-1.1801	0.08907	—	AT1G04240	IAA3

Continue

FPKM_ WT	FPKM_ *ysl*	Log2 Fold Change	Padj	Regulation	Gene	Gene name
18.59	20.92	-0.048553	0.93434	—	AT1G04550	IAA12
1.51	0.707	-1.3192	0.06414	—	AT1G15580	IAA5
29.43	34.47	0.013424	1	—	AT1G51950	IAA18
0.0596	0.0649	-0.084043	1	—	AT1G80390	IAA15
156.11	162.93	-0.15564	0.25443	—	AT2G22670	IAA8
17.23	20.64	0.04314	0.93942	—	AT2G33310	IAA13
0.331	0.297	-0.37443	0.94892	—	AT2G46990	IAA20
56.15	56.18	-0.21579	0.089532	—	AT3G04730	IAA16
16.39	18.76	-0.022683	1	—	AT3G16500	IAA26
0.0580	0.0927	0.47376	1	—	AT3G17600	IAA31
1.039	0.959	-0.33771	0.80816	—	AT3G62100	IAA30
158.04	195.73	0.093451	0.6713	—	AT3G23050	IAA7
6.78	6.96	-0.18076	0.59508	—	AT4G28640	IAA11
7.68	10.25	0.2009	0.69242	—	AT4G14550	IAA14
1.58	0.756	-1.2892	0.08662	—	AT4G32280	IAA29
25.91	26.02	-0.21149	0.41561	—	AT4G29080	IAA27
9.90	9.43	-0.28849	0.30689	—	AT5G25890	IAA28
50.69	56.66	-0.056754	0.81995	—	AT5G43700	IAA4
148.73	150.76	-0.1971	0.1092	—	AT5G65670	IAA9
5.38	5.05	-0.31099	0.23536	—	AT1G19850	MP
0.00725	0	—	1	—	AT1G34310	ARF12
23.65	30.22	0.13624	0.4036	—	AT1G59750	ARF1
21.11	21.67	-0.18101	0.26554	—	AT2G33860	ETT
9.37	12.91	0.24414	0.16038	—	AT3G61830	ARF18
8.94	9.41	-0.14263	0.52751	—	AT4G23980	ARF9
10.93	13.26	0.061626	0.82981	—	AT5G20730	NPH4
0	0.0137	—	0.97752	—	AT1G23160	T26J12.7
1.80	2.69	0.36012	0.32862	—	AT1G28130	GH3.17
0.195	0.178	-0.34629	0.91225	—	AT1G48660	F11I4.15
0.00706	0	—	1	—	AT1G48670	F11I4.14
0.0357	0	—	0.48902	—	AT2G14960	GH3.1
0.703	0.731	-0.16532	0.90255	—	AT2G47750	GH3.9
19.14	10.47	-1.083	0.20032	—	AT4G03400	DFL2
2.57	3.40	0.18505	0.67269	—	AT4G27260	WES1
0.779	0.572	-0.65934	0.21921	—	AT4G37390	BRU6
2.94	2.52	-0.43132	0.39947	—	AT5G13320	PBS3
6.94	9.43	0.22454	0.25772	—	AT5G13360	T22N19.10
0.104	0.116	-0.065114	1	—	AT5G13380	T22N19.30
2.90	3.53	0.064263	0.95994	—	AT5G51470	MFG13.8
3.51	7.11	0.80021	0.09415	—	AT1G16510	F3O9.31
1.70	2.13	0.10832	0.96655	—	AT1G19830	F14P1.18

Continue

FPKM_ WT	FPKM_ *ysl*	Log2 Fold Change	Padj	Regulation	Gene	Gene name
2.82	2.60	-0.33238	0.4854	—	AT1G19840	F6F9.11
2.59	4.36	0.53631	0.13473	—	AT1G75580	F10A5.21
1.17	1.40	0.041966	1	—	AT1G75590	F10A5.20
0	0.0193	—	1	—	AT1G79130	SAUR40
1.30	1.39	-0.12147	0.96109	—	AT2G16580	F1P15.4
0.247	0.337	0.22611	0.9949	—	AT2G18010	SAUR10
1.83	1.90	-0.16927	0.94959	—	AT2G21200	F26H11.4
0.942	0.655	-0.73513	0.40339	—	AT2G21220	F7O24.6
0.0396	0.157	1.7654	0.50658	—	AT2G24400	SAUR38
2.02	1.23	-0.92423	0.08482	—	AT2G28085	SAUR42
0.0501	0.101	0.79576	0.97762	—	AT2G36210	F2H17.18
0.115	0.100	-0.41195	1	—	AT2G37030	SAUR46
0.652	1.35	0.8442	0.16264	—	AT2G45210	SAUR36
15.38	16.06	-0.15638	0.60845	—	AT2G46690	SAUR32
1.15	1.20	-0.15178	0.994	—	AT3G09870	SAUR48
7.68	10.93	0.29525	0.25248	—	AT3G12830	SAUR72
1.88	1.21	-0.84708	0.35618	—	AT3G53250	SAUR57
1.46	1.93	0.18591	0.77763	—	AT3G20130	CYP705A22
0.532	0.286	-1.1253	0.58732	—	AT3G61900	SAUR33
0.0223	0	—	1	—	AT4G09530	SAUR17
0.0150	0	—	1	—	AT4G13790	F18A5.180
4.76	3.92	-0.50066	0.12727	—	AT4G00880	A_TM018A10
0.593	0.391	-0.82405	0.47685	—	AT4G22620	SAUR34
0	0.0136	—	1	—	AT4G31320	F8F16.140
20.14	21.21	-0.14069	0.8038	—	AT4G34750	F11I11.5
39.73	36.57	-0.33501	0.46639	—	AT4G34760	F11I11.11
1.52	1.88	0.091137	1	—	AT4G34800	F11I11.40
3.54	2.49	-0.72008	0.19021	—	AT4G34700	F11I11.10
0	0.0303	—	1	—	AT4G34780	F11I11.20
5.97	5.33	-0.37908	0.22324	—	AT4G34790	F11I11.30
1.23	1.46	0.024886	1	—	AT4G34810	F11I11.50
2.008	2.60	0.15903	0.90162	—	AT4G38850	SAUR15
23.07	32.65	0.28419	0.06927	—	AT3G60690	F19H22.1
0.643	0.208	-1.8371	0.05883	—	AT5G10990	SAUR69
1.27	0.279	-2.4174	0.29069	—	AT5G18010	SAUR19
5.63	4.18	-0.65418	0.4351	—	AT5G18020	SAUR20
4.26	3.18	-0.64608	0.44161	—	AT5G18030	MCM23.13
0.245	0.384	0.42531	0.85397	—	AT5G20810	SAUR70
0.191	0.0111	-4.3359	0.12921	—	AT5G50760	MFB16.16
0.198	0.115	-1.0212	0.79292	—	AT1G15050	IAA34
0	0	—	—	—	AT1G34390	ARF22

Continue

FPKM_ WT	FPKM_ *ysl*	Log2 Fold Change	Padj	Regulation	Gene	Gene name
0	0	—	—	—	AT1G35520	ARF15
0	0	—	—	—	AT1G35540	ARF14
0	0	—	—	—	AT1G59500	GH3.4
0	0	—	—	—	AT5G13350	T22N19.4
0	0	—	—	—	AT3G43120	SAUR39
0	0	—	—	—	AT3G51200	SAUR18
0	0	—	—	—	AT4G38825	SAUR13
0	0	—	—	—	AT5G66260	K1L20.4

Supplementary Table S4. SSLP makers for rough mapping.

Primer	Sequence
M1-F	GATATTTGTTTTGCTAACAC
M1-R	TAATAAAGTTCCAGCTTTGA
M2-F	CACTGCAACAAAGTGGAAAT
M2-R	ATCCGTTTCAATATCCACAA
M3-F	TGCGGGAGTGTGATAGAATA
M3-R	TCCTCGAAAGATTCATTGAT
M4-F	GAATTCTGTAACATCCCATTTCC
M4-R	GGTCTAATTGCCGTTGTTGC
M5-F	GGACCGACGGTTACGAGAGT
M5-R	TAACGGGCCGTTGCAAGA
M6-F	CGTGTTTACCGGGTCGGA
M6-R	AAAACCCTTGAAGAATACG
M7-F	TAGTCTGAGCTTACCAATA
M7-R	TTACCCTCGACTCGTAAC
M8-F	ATGTATTTGTTGCAAAATAA
M8-R	TGCACAGAAGAAAAAACTA
M9-F	TCCGATTCGATTAAACTC
M9-R	TTATTTCCTATTTCAAGACT
M10-F	ATGAACGGAGTAGCTATC
M10-R	CGCGTAGAACATAATCTGTA
M11-F	CAATGGGAAGAAGGTGTGAG
M11-R	CGCATTTCCATAAGTTTGTT
M12-F	ACCTGTTCAGTCTATGTTAC
M12-R	GGGAATTATTAACATTATCA
M13-F	ATGAGCTTTAGGAGTGTGTA
M13-R	AATTTTGTCCCAAAAGAATA
M14-F	CAAAAGAAATGCAACGAGAC
M14-R	TTTGATCATGAATGGTAGTG
M15-F	GAGCAACATTAAGGATAGAA
M15-R	ATCTCATACTCATAATATGTAG

M16-F	TTATAGCAAACGTACAAGTC
M16-R	CTGCATACACGTCGTCTC
M17-F	CTGGACCCTAGTGGATGT
M17-R	GACGGTTCTCCATTAATTAT
M18-F	TTCGGAGAAAGAAACGACAT
M18-R	ATGGAACTATTCAGGCATTA
M19-F	GCAACCGCTGCTGCTTTA
M19-R	AATATTTGGCTTTGCGTAGA
M20-F	ACCCTAAAACAATGTCTCTT
M20-R	TGCTAACATGGAAATTTGTC
M21-F	CTCTGTTGGGGCAAAACC
M21-R	GATGCTGGAGAGTAGCTTAG
M22-F	TTCATGAGAGCGGCATTC
M22-R	GCAAAATGTTTGGACAATTA
M23-F	CACAGGCCATTGGATGTA
M23-R	TGTTAGAACCCACCATTTG
M24-F	CCTGTTCCAATGAATATG
M24-R	TGTAGCTGCTGAGTTGTC
M25-F	AAAAGGCGACTACTAGCA
M25-R	GCCATTTATTTGGTCAAC

Supplementary Table S5. Primers used for vector construct or qPCR.

Primer	Sequence	Cloning Methods	Vectors
YSLrescue-F	GGGGACAAGTTTGTACAAAAAA GCAGGCTCGATGGTTT TCTACTTCAAGGCC	Gateway	pEarleyGate 101
YSLrescue-R	GGGGACCACTTTGTACAAGAAA GCTGGGTCCATGAAAT CGTCTTCAAGTTCTT		
YSL-CRISPR-1F	TAGGTCTCCCTACACCATCTTCAG TTTTAGAGCTAG		
YSL-CRISPR-2F	TAGGTCTCCTGAAGGTGTGCTGG GTTTTAGAGCTAG		
YSL-CRISPR-1R	CGGGTCTCAGTAGTCGCCATGCA CCAGCCGGG		
YSL-CRISPR-2R	CGGGTCTCATTCACTAATGTGCA CCAGCCGGG		
YSL-pro-F	GGGGACAAGTTTGTACAAAAAA GCAGGCTCGTCCCC TTCCAAATTTCTC	Gateway	pKGWFS7GUS
YSL-pro-R	GGGGACCACTTTGTACAAGAAA GCTGGGTCCGCTTC GATTCGAGAAAC		

GST-YSL-F	GGGGACAAGTTTGTACAAAAAA GCAGGCTCGATGGT TTTCTACTTCAAGGCC	Gateway	pDEST15
GST-YSL-R	GGGGACCACTTTGTACAAGAAA GCTGGGTCTTACATG AAATCGTCTTCAAGTTC		
HIS-VIK-F	GCGCGGCAGCCATATGATGAGCT CCGATTCACCGGC	NdeI BamHI	pET15
HIS-VIK-R	CAGCCGGATCCTCGAGTTATGAA GTGAATAAGCCCCA		
YSL-Yc-F	GGGGACAAGTTTGTACAAAAAA GCAGGCTTCATGGTT TTCTACTTCAAGGC	Gateway	Spyc
YSL-Yc-R	GGGGACCACTTTGTACAAGAAA GCTGGGTCCATGAAA TCGTCTTCAAGTT		
VIK-Yn-F	GGGGACAAGTTTGTACAAAAAA GCAGGCTTCATGAGC TCCGATTCACCGGC	Gateway	Spyn
VIK-Yn-R	GGGGACCACTTTGTACAAGAAA GCTGGGTCTGAAGTG AATAAGCCCCAAT		
H2A.Z-CFP-F	GGGGACAAGTTTGTACAAAAAA GCAGGCTTCATGGCTGGTAAAGG TGGGAA		
H2A.Z-CFP-R	GGGGACCACTTTGTACAAGAAA GCTGGGTCATCCTTGGTGACTTT GTTGA		
PIP2A-mCherry-F	GGGGACAAGTTTGTACAAAAAA GCAGGCTTCATGGCAAAGGATGT GGAAGC		
PIP2A-mCherry-R	GGGGACCACTTTGTACAAGAAA GCTGGGTCGACGTTGGCAGCACT TCTGA		
ACTIN2-qF	CCCGATGGGCAAGTCATC		
ACTIN2-qR	GAACAAGACTTCTGGGCATCTGA		
YSL-qF	AAGGGCAGAGAGGAAACAACAT C		
YSL-qR	TGCCTCTCCTTCTCAAGCCTAT		
VIK-qF	GGGCGGTCTTACTCCAACAA		
VIK-qR	CATTCCTCTCGCGATATCCAA		
IAA19-qF	AAGCTTCGACCACGAAAGTG		
IAA19-qR	ACCATCTTTCAAGGCCACAC		
SAUR9-qF	AAAAGGTCACTTCCCGGTCT		

SAUR9-qR	CTCGGCTAGTTGGAGGAGTG
SAUR22-qF	CCAAAAGGGTTTCTTGCAGT
SAUR22-qR	ATCGGATGATCAAACCCAAA
SAUR23-qF	CCAAAAGGGTTTCTTGCAGT
SAUR23-qR	CGAACCCAAACTCTTCTTCG
SAUR27-qF	TTCAGCACCAAAAGGGTTTC
SAUR27-qR	CGGATGATCAAATCCGAACT
